# Spatial modelling of the infestation indices of *Aedes aegypti*: an innovative strategy for vector control actions in developing countries

**DOI:** 10.1186/s13071-020-04070-w

**Published:** 2020-04-16

**Authors:** Ana Carolina Policarpo Cavalcante, Ricardo Alves de Olinda, Alexandrino Gomes, John Traxler, Matt Smith, Silvana Santos

**Affiliations:** 1 Public Health Program, Universidade Estadual da Paraíba, Campina Grande, Paraíba CEP 58429-500 Brazil; 2 University of Wolverhampton, Institute of Education, Walsall Campus, Gorway Road, Walsall, WS1 3BD UK

**Keywords:** Arbovirus infections, *Aedes*, Spatial analysis

## Abstract

**Background:**

Larval indices such as the house index (HI), Breteau index (BI) and container index (CI) are widely used to interpret arbovirus vector density in surveillance programmes. However, the use of such data as an alarm signal is rarely considered consciously when planning programmes. The present study aims to investigate the spatial distribution pattern of the infestation of *Aedes aegypti*, considering the data available in the *Ae. aegypti* Infestation Index Rapid Survey (LIRAa) for the city of Campina Grande, Paraíba State in Brazil.

**Methods:**

The global and local Moranʼs indices were used in spatial analysis to measure the effects of spatial dependencies between neighbourhoods, using secondary data related to HI and BI gathered from surveillance service.

**Results:**

Our analysis shows that there is a predominance of high rates of mosquito infestation, placing Campina Grande at a near-constant risk of arbovirus outbreaks and epidemics. A highly significant Moranʼs index value (*P* < 0.001) was observed, indicating a positive spatial dependency between the neighbourhoods in Campina Grande. Using the Moran mapping and LISA mapping, the autocorrelation patterns of *Ae. aegypti* infestation rates among neighbourhoods have revealed hotpots that should be considered a priority to preventive actions of the entomological surveillance services. Predominance of high infestation rates and clearer relationships of these between neighbourhoods were observed between the months of May and July, the period with the highest rainfall in the city.

**Conclusions:**

This analysis is an innovative strategy capable of providing detailed information on infestation locations to the relevant public health authorities, which will enable a more efficient allocation of resources, particularly for arbovirus prevention.
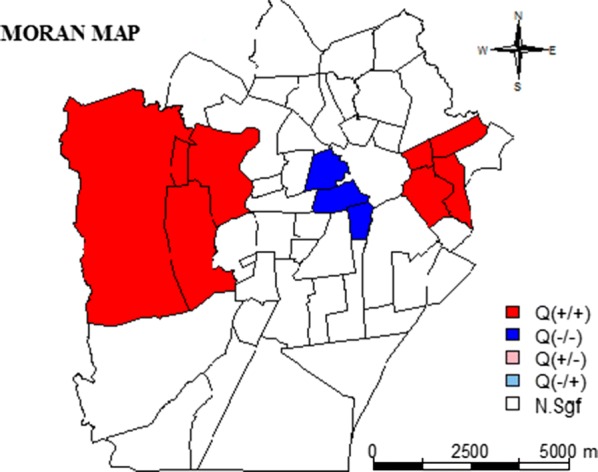

## Background

The control of *Aedes aegypti*, a widespread vector of several viruses, is still considered a major challenge for public health authorities, especially in developing countries. In Brazil, epidemics of the diseases transmitted by *Ae. aegypti*, i.e. dengue, chikungunya and Zika, have been responsible for significant human and economic losses, and led the authorities to formulate strategies to attempt to increase vector control [1]. In 2015 and 2016, an annual average of 1,586,155 probable cases of dengue were recorded in Brazil; although a reduction to 252,054 cases was observed in 2017 [2], which could be the result of the cross-herd immunity to Zika virus [3]. The differential diagnosis of these diseases is difficult because of the similarity of symptoms, cross-reactivity, co-circulation and overlap of infections by different arbovirus species and strains [4, 5]

Arboviral transmission has multiple causes and contributory contextual factors, impacted by the influence of social, environmental and cultural determinants in the course of the natural history of disease [4]. Epidemiological studies have clearly associated the expansion of these diseases with haphazard and unplanned urban development processes, mainly caused by the lack of infrastructure and basic sanitation in areas that have been occupied without prior planning. In north-east Brazil, the population has been disproportionately affected by arboviruses, with 94% of all cases of congenital Zika syndrome being reported in this region [2, 6, 7]. Poor access to garbage collection and an intermittent water supply provide conditions for vector breeding, which in turn makes vector control more difficult in this region [3].

In Brazil and elsewhere, arbovirus control programmes preferentially use survey of larval indices of *Ae. aegypti* population assessment, owing to practicality and reproducibility [8, 9]. To identify the most vulnerable areas and provide indices rapid and timely, the *Ae. aegypti* Infestation Index Rapid Survey (LIRAa) has been applied by municipalities in Brazil since 2003 in accordance with the guidelines of National Dengue Control Programme (PNCD) [10, 11].

For LIRAa, the municipality is divided into strata of 8100–12,000 individuals; sampling is performed in two phases by blocks of houses (primary sampling unit) and by houses (secondary sampling unit) with a maximum sample limit of 450 houses [12]. Software is used to generate a list of starting blocks and subsequent blocks are selected according to a sampling interval. In each selected block, endemic disease control agents (ACEs) survey an average of 25 houses per day, targeting every fifth house on a street (i.e. 20% of properties). They collect information on infested containers, which are classified in different categories (containers for domestic water separation, mobile, fixed, discarded and natural containers) [12].

The presence or absence of dengue vectors is measured using three key indices: house index (HI: the percentage of houses infested with larvae and/or pupae); Breteau index (BI: the number of positive containers per 100 houses); container index (CI: the percentage of water-holding containers infested with larvae or pupae) [12, 13]. These indices may facilitate understanding of the ecology of vectors in a given control area, but they also serve as useful measures for determining the success of intervention strategies [11, 14]. In Brazil, data for the calculation of infestation indices is collected on a bimonthly basis according to PNCD guidelines [12]. However, some flexibility is given to the municipality surveillance services, which are usually performed four times a year, but increase their frequency during outbreaks and epidemic [8, 9].

According to the PNCD, HI values above 4% indicate risk of dengue outbreak; above 1% and below 3.9%, alert situation; and below or equal 1%, satisfactory situation. BI above 5 indicates a risk situation. These thresholds are currently used by the surveillance services to plan and execute preventive actions, such as spraying insecticides in some neighbourhoods. However, in the literature, there is no evidence of correlation between larval density and disease transmission. It means that these thresholds cannot be useful to predict outbreaks [15].

Spatial analysis allows the identification and explanation of the geographical distribution of disease patterns. It comprises the quantitative study of phenomena that are geographically located in space and may be performed by visualization, exploratory analysis or spatial data modelling methods. This type of approach has been widely used in epidemiological studies and allows for an exploration of the relationship between demographic, environmental and socioeconomic information in order to detect the conditions and determinants of the arboviruses without dissociating them from the context of their territorial spaces [16].

In this study, the spatial and temporal distribution patterns of infestation of *Ae. aegypti* were investigated, using LIRAa data from the period 2014 to 2017 for Campina Grande City, Brazil. This was completed in order to evaluate the potential of using the autocorrelation statistics between neighbourhoods as an infestation indicator. Moreover, the concept of strata within the LIRAa data was devised and investigated for their potential contribution to the targeting of, and possibly greater effectiveness of, vector control actions.

## Methods

This study evaluated secondary data from the house Index (HI) and the Breteau index (BI), both gathered by the surveillance services. The units of analysis were 51 neighbourhoods, grouped in 17 strata, of the city of Campina Grande, Brazil, from 2014 to 2017. This period was delimited considering the hypothesis that Zika virus were introduced in Brazil during the 2014 FIFA World Cup [17]. The team from Tahiti (French Polynesia) had played in the Pernambuco Arena in June 2013 and the viral phylogenetic study showed that the origin of the Brazilian strain was Asian, sharing a common ancestor circulating in French Polynesia [18]. The Zika virus may have probably been introduced in Pernambuco, which could explain the larger size of the epidemic in this state and neighbouring areas, such as Campina Grande [19]. Zika virus was associated with a high prevalence of cases of congenital syndrome Zika, which has led the country to an emergency public health situation [20].

Campina Grande city (7°13′14.92″S, 35°55′1.32″W) is considered one of the main industrial centres of the north-east of Brazil as well as the main technological core within South America. In 2019, its estimated population was 409,731 inhabitants, making it the second-most populous city of Paraíba, with a population density of 648.31 per km^2^ [21]. About 40% of the population exists on a very low wage, or about USD 118 per month (half of the minimum wage) [21].

Based on the Köppen-Geiger climate classification system, Campina Grande has a moderate tropical climate, with a dry season from September to January and a wet season from May to August. Maximum summer and winter temperatures are 30 and 18 °C, respectively. Minimum summer and winter temperatures are 20 and 15 °C, respectively. The annual relative humidity is between 75–82% [22].

**Fig. 1:**
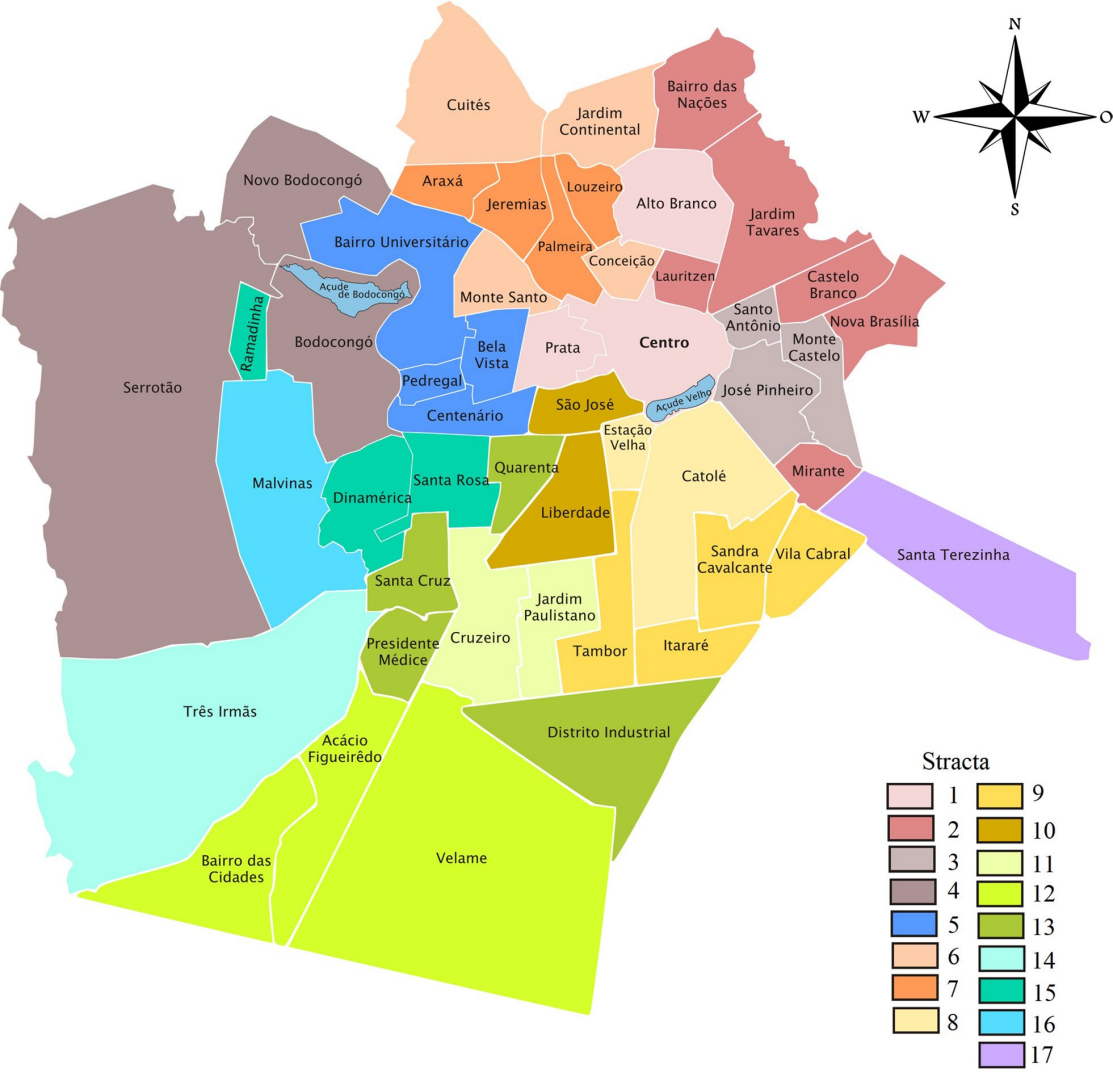
Map of Campina Grande city, Paraiba State, Brazil showing 17 strata in which are grouped together 51 neighbourhoods

The city has a total area of 593 km^2^, divided into 51 neighbourhoods with 5% designated as rural and 95% as urban (Fig. 1). Much of the city’s growth was not planned, and its neighbourhoods correspond to old farms that were sold and urbanized. The poorer neighbourhoods correspond to the peripheral areas or those near rivers or railroads. From 2014 to 2017, the cityʼs neighbourhoods were grouped into 17 strata, which were grouped together taking into consideration socioeconomic characteristics and/or physical factors, such as large avenues, highways, railways, wide water flows such as rivers, lakes and dams. All procedures used to define strata are described in guidelines published by the Brazilian Ministry of Health, which also provides software for sampling and collecting data [10].

### Statistical methods

For the purposes of this study, local authorities of Campina Grande provided the results of the LIRAa (HI and BI), which was carried out between January 2014 and December 2017. The dependent variables were 51 records of HI and BI, collected three to five times per year. The main descriptive statistics for these dependent variables are presented in the results: maximum, minimum, median, interquartile intervals, mean and standard deviation. For spatial visualization of the data, the quartile maps, Moran map and Local Indicators of Spatial Association (LISA) map are presented for 2014 and 2017. The other years are presented in Additional file 1: Figure S1, Additional file 2: Figure S2, Additional file 3: Figure S3, Additional file 4: Figure S4, Additional file 5: Figure S5, Additional file 6: Figure S6, Additional file 7: Figure S7, Additional file 8: Figure S8, Additional file 9: Figure S9 and Additional file 10: Figure S10. All analyses were performed using the R software [23].

### Matrix W

The associations of neighbourhood observations, defined for each location, can be expressed by spatial contiguity or a weight matrix W of order n × n, where n is the number of locations (neighbourhoods). The entry in the ith row and jth column, denoted as W_ij_, corresponds to the pair (i, j) of locations. The elements of the matrix W_ij_ assume a nonzero value when the areas (observations) i and j are considered neighbouring, and zero otherwise.

$$W = \left[ {\begin{array}{*{20}c} {W_{11} } & {W_{12} } & \cdots & {W_{1n} } \\ {W_{21} } & {W_{21} } & \cdots & {W_{2n} } \\ \vdots & \vdots & \ddots & \vdots \\ {W_{n1} } & {W_{n2} } & \cdots & {W_{nn} } \\ \end{array} } \right]$$

### Spatial autocorrelation

Spatial correlation is the correlation between observations of a single variable solely attributable to their proximity in space. Spatial autocorrelation (association) measurements and tests can be differentiated by the range or scale of analysis, as distinguished from global and local measures [24]. A global measure implies that all elements in the matrix W are included in the spatial correlation calculation, producing a spatial autocorrelation value for any spatial weight matrix. In contrast, local measures are concentrated, i.e. they evaluate the autocorrelation associated with one particular area or a few area units rather than all of them [24].

Both measures indicate the degree of spatial association of the dataset. The Moranʼs I index calculates the spatial autocorrelation as a covariance, from the product of the deviations from the mean [24]. This index indicates the magnitude of the spatial association present in the data set with n locations. The Moranʼs I index is calculated by the following expression:

$$I = \frac{{\sum\nolimits_{i = 1}^{n} {\sum\nolimits_{j = 1}^{n} {w_{ij} \left( {y_{i} - \bar{y}} \right)\left( {y_{j} - \bar{y}} \right)} } }}{{\sum\nolimits_{i = 1}^{n} {\left( {y_{i} - \bar{y}} \right)^{2} } }}$$

The Moranʼs I index varies in a range of (− 1:1), where − 1 means perfect dispersion, 0 represents random behaviour, and 1 means perfect association. Assuming that Z_i_ is observations of random variables Z_i_ whose distribution is normal, then it has an appropriately normal distribution:

$$E(I) = - \frac{1}{(n - 1)}$$

$$Var(I) = \frac{{n^{2} (n - 1)W_{1} - n(n - 1)W_{2} - 2W_{0}^{2} }}{{(n + 1)(n - 1)^{2} W_{0}^{2} }}$$

While these comprehensive measures are very useful to provide an indication of global grouping data, such methods need to be complemented by local statistics. The formula for calculating the local Moranʼs index for each area A_i_ is given by:

$$I_{i} = \frac{{(y_{i} - \bar{y})\sum\nolimits_{i = 1}^{n} {\sum\nolimits_{j = 1}^{n} {w_{ij} \left( {y_{i} - \bar{y}} \right)} } }}{{\sum\nolimits_{i = 1}^{n} {\frac{{\left( {y_{j} - \bar{y}} \right)^{2} }}{n}} }}$$

The statistics can be interpreted as follows: positive values of I_i_ mean that there are spatial clusters with similar values (high or low) of the variable under study, whereas negative values mean that there are spatial clusters with dissimilar values of the variable in and between the areas and their neighbours.

### Moran scatter plot

The Moran scatter plot is an illustration of the relationship between the values of the chosen attribute at each location and the average value of the same attribute at neighbouring locations. For this purpose, the diagram is divided into four quadrants (Q1, Q2, Q3 and Q4) with the following interpretation: (i) Q1: the first quadrant (upper right) shows the areas that have high values for the variable in question surrounded by neighbouring areas which also have above-average values for the variable under analysis. This quadrant is classified as high-high (AA, + +); (ii) Q2: the second quadrant (lower left) shows the areas that have low values for the variable in question surrounded by neighbouring areas that also have below-average values for the analysed variables. This quadrant is classified as low-low (BB, − −); (iii) Q3: the third quadrant (lower right) shows the areas that have high values for the variable under analysis surrounded by neighbouring areas that have values below the average for the variable in question. This quadrant is classified as high-low (AB, + −); and (iv) Q4: the fourth quadrant (upper left) shows areas that have low values for the variable under analysis surrounded by areas that are above the average for the variable in question. This quadrant is classified as low-high (BA, − +).

The areas located in quadrants Q1 and Q2 show positive autocorrelation, i.e. the neighbouring areas had similar value. In contrast, the areas located in quadrants Q3 and Q4 have negative autocorrelation, i.e. there is dissimilarity between the neighbouring areas.

### Box map, LISA map and Moran map

The Box map is an extension of the Moran scatterplot in which the elements of each quadrant of the plot are represented by a specific colour with their respective polygons. The LISA map indicates the regions whose location correlation is significantly different from the others, being classified into the following groups: non-significant; and significant at the 5% (*P* < 0.05), 1% (*P* < 0.01), and 0.1% (*P* < 0.001) levels, respectively. The Moran map, similarly to the LISA map, shows only significant values, being represented in four groups and placed in the quadrants to which they belong on the graph.

## Results

Table 1 shows the descriptive analysis of data through the period 2014 to 2017. The HI (*n* = 51) ranged from 0.30 to 21.50 with an average of 4.74 ± 2.10; while the BI (*n* = 51) ranged from 0.30 to 21.20 with an average of 4.96 ± 2.17. The mean values observed for HI were greater than 4%, and for BI were very close to 5, indicating risk of outbreaks and epidemics. It is noteworthy that the BI was almost exactly consistent with the HI predictions.

**Table 1:** Descriptive analysis of *Aedes aegypti* Rapid Index Survey (LIRAa) measurements, Breteau Index (BI) and House Index (HI), during the period of 2014–2017, in Campina Grande, Paraíba, Brazil

Year	Month	*n*	Breteau Index					House Index				
	LIRAa		Range	Median	Q1	Q3	Mean ± SD	Range	Median	Q1	Q3	Mean ± SD
2014	1. January	51	0.3–4.4	1.3	1.00	2.5	1.84 ± 1.15	0.3–4.4	1.3	1.0	2.5	1.80 ± 1.15
	2. March	51	0.5–7.9	1.8	1.00	2.7	2.16 ± 1.48	0.5–7.7	1.8	1.0	2.6	2.08 ± 1.43
	3. May	51	0.3–10.2	3.2	2.3	4.3	3.54 ± 1.93	0.3–10.2	3.2	2.3	4.0	3.40 ± 1.84
	4. July	51	1.0–11.4	4.0	2.7	5.9	4.57 ± 2.76	1.00–11.4	3.6	2.5	5.7	4.14 ± 2.46
	5. October	51	0.9–7.0	3.3	2.6	3.9	3.02 ± 1.12	0.9–7.0	3.3	2.2	3.9	2.93 ± 1.17
2015	1. January	51	1.7–12.1	5.0	3.4	5.6	4.86 ± 1.78	1.7–10.9	4.6	3.1	5.5	4.37 ± 1.68
	2. March	51	0.4–12.5	4.8	3.8	6.5	5.14 ± 2.27	0.4–12.5	4.8	3.8	5.4	4.86 ± 2.20
	4. July	51	3.7–20.5	7.8	6.4	9.7	8.31 ± 2.97	3.7–20.0	7.8	5.7	9.7	7.83 ± 2.83
	5. October	51	2.6–17.5	5.0	4.6	8.3	6.48 ± 2.92	2.6–17.2	5.0	4.4	7.5	6.25 ± 2.81
2016	1. April	51	2.8–17.3	7.1	4.7	8.1	6.79 ± 2.36	2.6–17.0	7.1	4.6	8.0	6.57 ± 2.30
	2. July	51	2.5–9.3	4.5	3.3	6.2	4.61 ± 1.50	2.5–9.3	4.5	3.1	6.2	4.50 ± 1.54
	3. October	51	1.2–6.0	2.1	1.8	2.9	2.59 ± 1.25	1.2–5.5	2.1	1.7	2.9	2.50 ± 1.26
2017	1. January	51	2.9–21.5	7.8	5.3	10.3	7.65 ± 3.56	2.9–21.5	6.8	4.7	10.0	7.36 ± 3.55
	2. April	51	0.7–11.8	4.3	3.8	6.0	5.15 ± 2.39	0.7–11.6	4.3	3.8	6.0	5.06 ± 2.31
	3. July	51	1.8–21.5	7.5	6.9	9.3	7.70 ± 3.13	1.8–21.5	7.5	6.3	9.0	7.40 ± 3.00

*Abbreviations*: n, number of observations; MD – median; Q1, first quartile; Q3, third quartile; SD, standard deviation

According to the global Moranʼs index (W), a significant spatial autocorrelation among the analysed neighbourhoods (*n* = 51) for the most of dependent variables (HI and BI) was observed with *P* < 0.05 (Table 2), during the period 2014–2017. This result indicates that there is a clear spatial dependence in the mosquito infestation proportion among neighbourhoods, demonstrating that those in close proximity have a greater risk of cross-infestation (Table 2).

**Table 2:** Global Moranʼs index 2014–2017

Variable	2014		2015		2016		2017	
	Moran	*P*	Moran	*P*	Moran	*P*	Moran	*P*
BI-1	0.3689	< 0.001	0.1978	< 0.001	0.0801	0.1168	0.1818	< 0.001
BI-2	0.3027	< 0.001	− 0.0414	0.5972	0.3402	< 0.001	0.0171	0.3355
BI-3	0.3281	< 0.001	0.1638	0.0155	0.2845	< 0.001	0108	0.0635
BI-4	0.2075	< 0.001	0.1782	0.0107	–	–	–	–
BI-5	− 0.0008	0.4127	–	–	–	–	–	–
HI-1	0.3877	< 0.001	0.0633	0.1667	0.0725	0.1345	0.2026	< 0.001
HI-2	0298	< 0.001	− 0.0281	0.5374	0.3532	< 0.001	0.0153	0.3435
HI-3	0.3147	< 0.001	0.1763	< 0.001	0.3045	< 0.001	0.0969	0.0777
HI-4	0.2501	< 0.001	0.1862	< 0.001	–	–	–	–
HI-5	0.0354	0.2627	–	–	–	–	–	–

*Note*: The Moran index and *P*-value (*P*) are shown for Breteau index (BI) and house index (HI)

Data from the HI and BI of the first LIRAa 2014 (January) showed that of the 51 neighbourhoods of the city studied, 9 had low-risk with HI less than 0.9%, 37 had an average risk with HI of 1–3.9%, and 4 were at high risk for outbreaks and epidemics by arboviruses, with HI greater than 4.0% (Galante, Mirante, Monte Castelo and Santo Antonio). In the second LIRAa of 2014 (March), the number of neighbourhoods at high risk of outbreak increased to eight and there was a change in the geographic distribution pattern (Tambor, Itararé, Bodocongó, New Bodocongó, Malvinas, Sandra Cavalcante, Serrotão and Vila Cabral).

From March to June, there was a considerable increase in neighbourhoods with a high risk, which increased from 5 in January to 22 neighbourhoods in June, with infestation predominating in the northern and western areas, such as the neighbourhoods of Mirante, Monte Castelo and São José. Given this situation, the municipality had an average infestation rate of 4.0%, which was considered as a high alert warning due to the high risk of outbreaks and epidemics arboviruses.

**Fig. 2:**
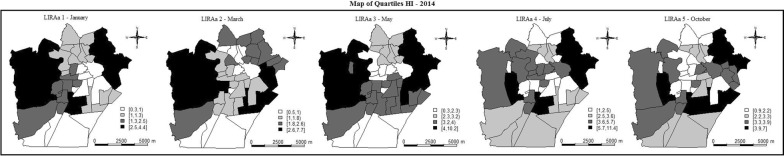
Moranʼs index map (Map of Quartiles) for the house index (HI) showing the autocorrelation of *Aedes aegypti* mosquito infestation between neighbourhoods of Campina Grande city, Paraiba State, Brazil, in 2014

**Fig. 3:**
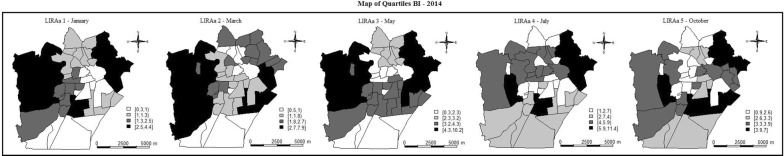
Moranʼs index map (Map of Quartiles) for the Breteau index (BI) showing the autocorrelation of *Aedes aegypti* mosquito infestation between neighbourhoods of Campina Grande city, Paraiba State, Brazil, in 2014

The autocorrelation of *Ae. aegypti* mosquito infestation between the city neighbourhoods studied can be identified by the Global Moran index map (map of quartiles) for HI and BI (Figs. 2 and 3, respectively) which shows the similarity in terms of the infestation of neighbouring areas. The positive values of the LIRAa shown by the increasingly dark shades used on the map indicate the clusters with similar spatial autocorrelation values and with statistical significance. Negative values on the LIRAa are represented by white, showing that there are dissimilar spatial clusters between contiguous areas. On the map, there is a prevalence of darker shades, indicating that most neighbourhoods have similar infestation values. The neighbourhoods with positive correlation did not necessarily correspond to those which belong to the same stratum based on similar LIRAa values indicating that the neighbourhoods belonging to the same stratum are not necessarily autocorrelated.

In 2015, there was a worsening of *Ae. aegypti* mosquito infestation rates. On the first LIRAa of the year, held in January, 34 neighbourhoods had an HI greater than 4.0%, i.e. more than half of the municipality was at high risk of an outbreak. In the second LIRAa, only one neighbourhood located in the city centre had a satisfactory index, i.e. less than 1%. Over time, the number of neighbourhoods with high infestation rate dropped from 34 to 30, but the value of this indicator rose in almost every neighbourhood, which resulted in an increase in the overall index of the municipality, which rose from 4.4% to 4.9%.

As in the previous year, the survey conducted in July 2015 (the third LIRAa) reached the highest levels of infestation, with 49 neighbourhoods at high risk and only three at medium risk. Very high levels of infestation were detected in the neighbourhoods of Presidente Medici (11.5%), Cruzeiro (11%) and Jardim Paulistano (11%), all belonging to the east zone. In the last survey of 2015 (the fourth LIRAa), there was a small reduction in the number of neighbourhoods with high risk, from 49 to 44. The Moranʼs index map for HI and BI in 2015 (Additional file 1: Figure S1, Additional file 2: Figure S2, respectively) shows the similarity of neighbouring areas with statistically significant autocorrelation.

In 2016, more than half of the city’s municipal neighbourhoods presented high levels of infestation, with an overall rate of 4.3%. Due to the strike of endemic disease control agents of 2016, only three surveys were conducted. On third LIRAa, the last of the year, held in October, it can be seen that there was a significant improvement in the rates when compared with other surveys of the same year. However, none of the neighbourhoods reached a satisfactory index, i.e. low risk, with the majority remaining on high alert, with medium risk. The Moranʼs index map for HI and BI in 2016 (Additional file 3: Figure S3, Additional file 4: Figure S4, respectively) highlights the similarity of the infestation pattern in adjoining neighbourhoods, as already shown for 2014 and 2015.

In 2017, approximately 81% of the neighbourhoods were at high risk in the first LIRAa (February). There was an improvement in indicators in the second LIRAa, but still approximately 65% of neighbourhoods were at high risk, 30% at medium risk and only 5% at low risk, culminating in an overall infestation rate of 4.9%. The final survey of the year, held in July, showed a significant increase again in infestation levels: back up to 87% of neighbourhoods at high risk and 13% at medium risk. The neighbourhoods of Malvinas, President Medici and Cruzeiro remained with a high level of infestation across all the different measurements (10%).

**Fig. 4:**
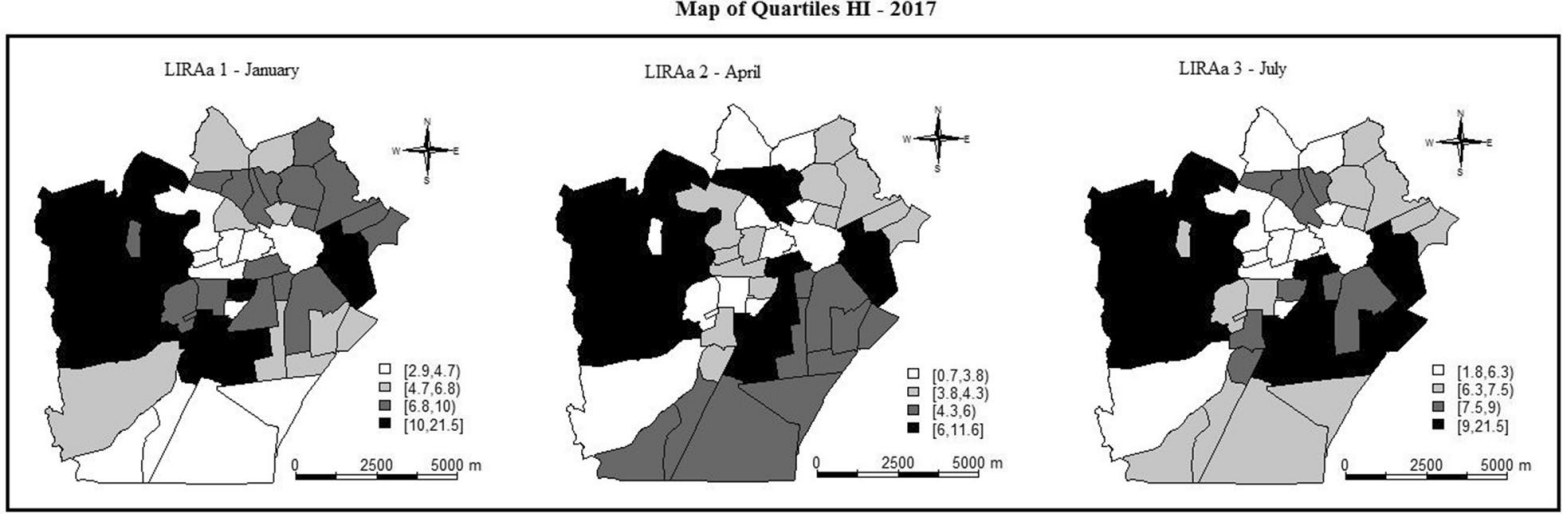
Moranʼs index map for the house index (HI) showing the autocorrelation of *Aedes aegypti* mosquito infestation between neighbourhoods of Campina Grande city, Paraiba State, Brazil, in 2017. The dark shades indicate the clusters with similar spatial autocorrelation values and with statistical significance

**Fig. 5:**
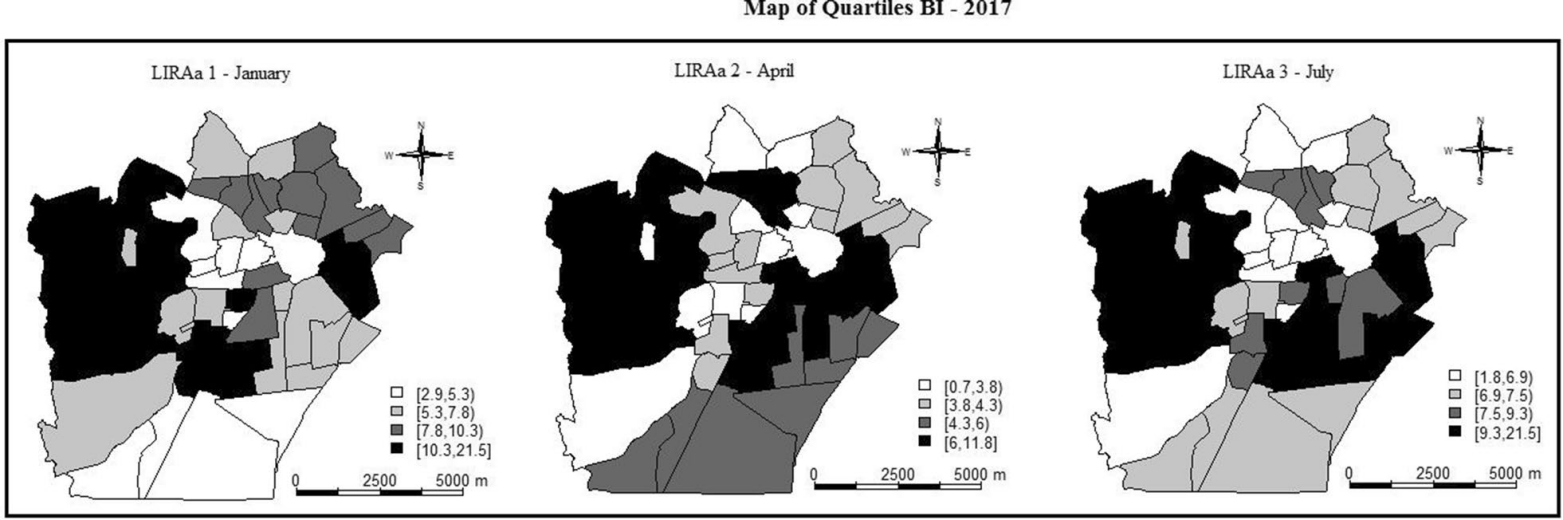
Moranʼs index map for the Breteau index (BI) showing the autocorrelation of *Aedes aegypti* mosquito infestation between neighbourhoods of Campina Grande city, Paraiba State, Brazil, in 2017. The dark shades indicate the clusters with similar spatial autocorrelation values and with statistical significance

Figures 4 and 5 present the global Moran map index (map of quartiles) for HI and BI, respectively, for three LIRAs performed in 2017. The dark shades indicate the clusters with similar spatial autocorrelation values and with statistical significance. It is possible to observe a repetition in the pattern of autocorrelated areas throughout the year.

**Fig. 6:**
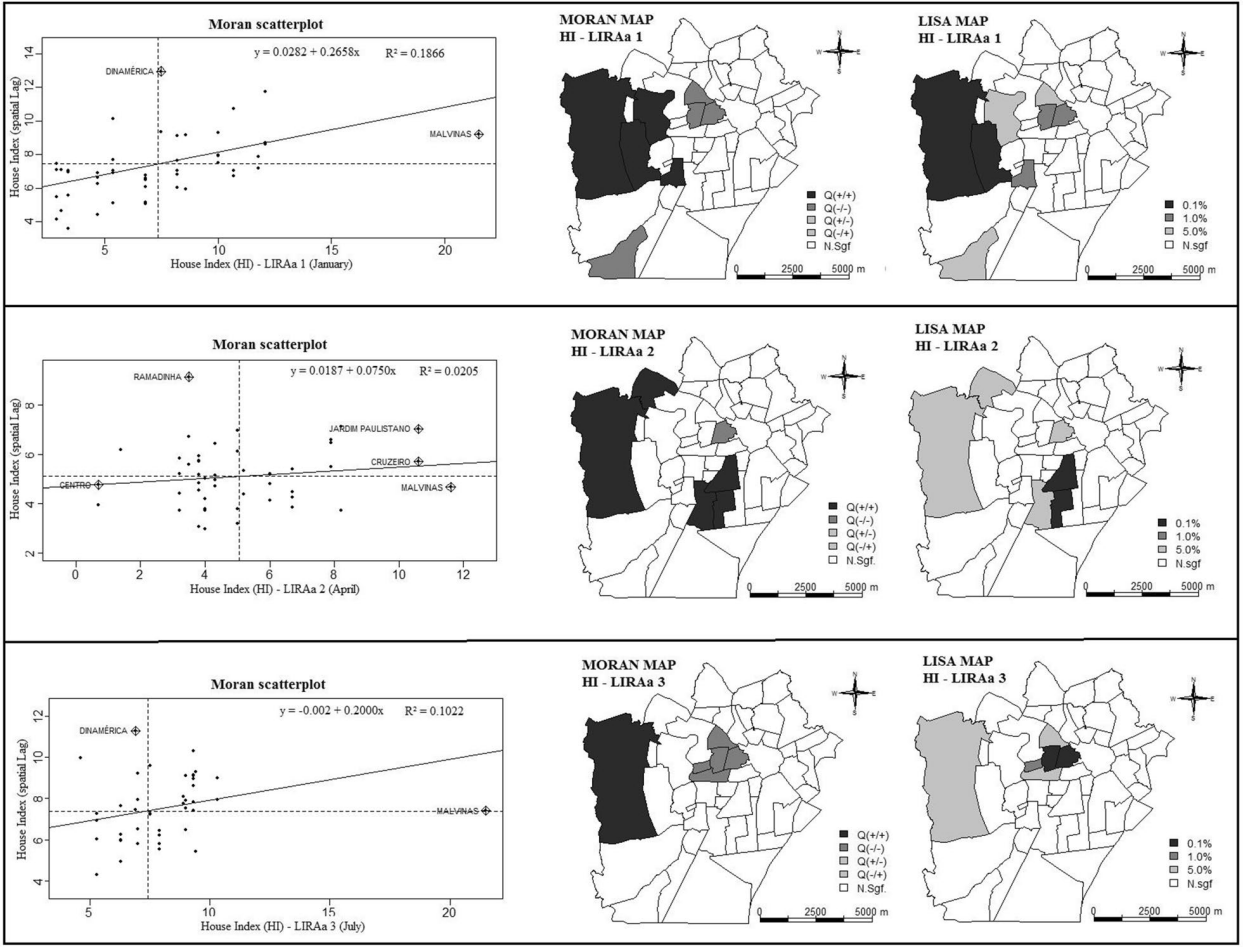
Moran scatterplots of the HI data, the Local Indicators of Spatial Association (LISA) maps, and the Moran maps in 2017

**Fig. 7:**
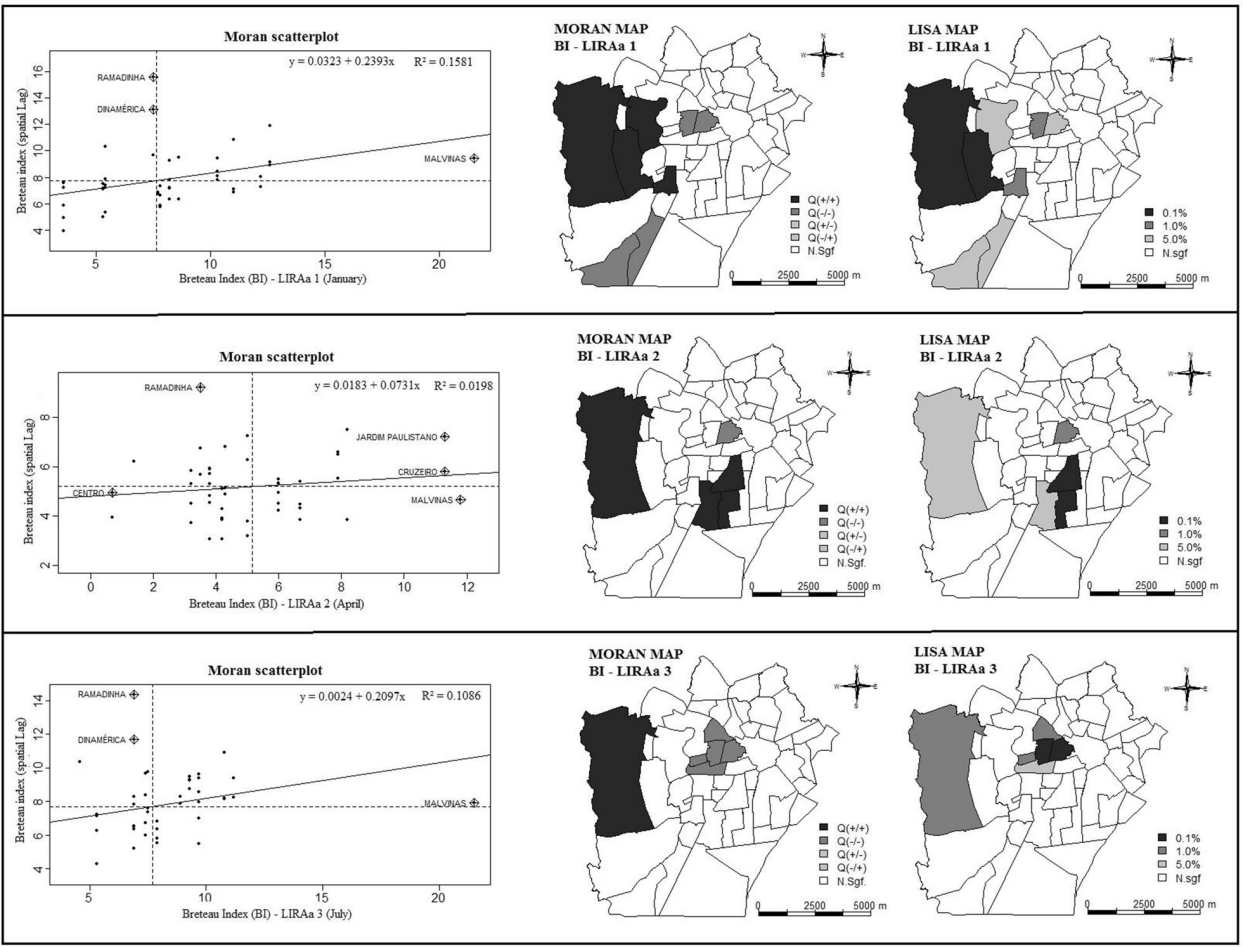
Moran scatterplots of the BI data, the Local Indicators of Spatial Association (LISA) maps, and the Moran maps in 2017

Figures 6 and 7 showed the Moran scatterplots, the LISA maps, and the Moran maps for HI and BI obtained in 2017. The scatterplots demonstrate the localities that were in the +/+ quadrant (i.e. showed a positive correlation with mosquito infestation) and these are shown on the Moran maps in black. The neighbourhoods in the −/− quadrant, shown in grey, are those with a negative value in the LIRAa data. The Moran maps directly represent the observations on the scatterplot onto the urban map. For example, in Fig. 6 (HI for 2017), Malvinas (in black), which comprises one of the poorest populations of the Campina Grande city, exerts a positive influence on the indicators. The LISA maps for each LIRAa survey show the alpha significance values, ranging from non-significant (white) to significant values (5% in light grey and 0.1% in black). The neighbourhoods in black are those that had the most significant influence on other localities.

Analysis of the scatterplots and the Moran and LISA maps for the period of 2014 up to 2017 showed that there is a clear repetition of patterns through time and that some neighbourhoods have a higher incidence of infestation rates, such as Malvinas, Ramadinha, José Pinheiro and Santo Antônio (Additional file 5: Figure S5, Additional file 6: Figure S6, Additional file 7: Figure S7, Additional file 8: Figure S8, Additional file 9: Figure S9 and Additional file 10: Figure S10). The use of this methodology thus reveals the hotspots or neighbourhoods that should be considered a priority for the preventive actions of the entomological surveillance services.

## Discussion

This is the first study to show the spatial autocorrelation of *Ae. aegypti* mosquito infestation among the neighbourhoods of Campina Grande. The results demonstrate autocorrelation distribution patterns evolving throughout the year. This analysis is an innovative strategy and may be more effective in planning surveillance actions than infestation rates, as suggested by the literature [25]. Spatial autocorrelation more accurately points out localities with similar levels of vulnerability and risk of *Ae. aegypti* infestation, which may help direct the actions of the responsible health authorities.

Spatial analysis has been used to show correlations between entomological indicators with the climate, disease transmission and socioeconomic conditions [26–28]; however, the strategy had not yet been used to analyse contiguous, or adjoining, neighbourhoods in order to better define areas of similar risk and vulnerability. Data from the literature highlight that the results of scientific research have not impacted the vector control practices in many countries, including Brazil [3, 8] However, the methodological approach and results of the present study are promising and can be easily replicated in future entomological surveillance services.

In Campina Grande, there was a predominance of high rates of mosquito infestation in different years indicating risk situations for outbreaks and epidemics. A similar study conducted in three cities in Kenya in order to assess the potential risk for the transmission of dengue and yellow fever, considered both BI and CI indices, as well as the seasonality of these regions. Based on the established vector index thresholds, the results have shown low to medium risk levels for urban yellow fever and high risk for dengue in Kilifi and Kisumu, while for Nairobi the yellow fever risk was lower and dengue risk levels were low to medium [28].

Other studies conducted in Taiwan, Sri Lanka and Vietnam [13, 14, 29] have shown that larval indices may be closely related to the incidence of dengue epidemics and arboviruses. In Brazil, similar research was conducted in Rio de Janeiro, São Paulo and Maranhão [14, 18, 19]. These studies used the infestation index in isolation; this differs from the present study which shows that patterns of spatial distribution and risk levels from small territorial units of analysis (census tracts, for example), can indicate that these risks can be connected into larger units, especially amongst adjoining neighbourhoods.

Some studies have examined land use and larval density. In Thailand, it has been observed that settlements around gas stations and workshops nearby swampland, marshes and paddy fields appear to be favourable habitats for vector propagation [30]. In Ethiopia, the most common *Ae. aegypti* mosquito breeding habitats were discarded tires (57.5%), followed by clay pots (30.0%) [31]. The approach here described may show how these favourable habitats for mosquito proliferation impact on contiguous areas, helping to redefine the risk territories and allow for a better direction of surveillance and resourcing to combat the disease vectors.

The literature describes contextual factors such as climate, seasonality and socioeconomic conditions for disease vector control. A survey conducted in Taiwan, for example, observed whether low temperatures can influence the distribution of the mosquito and identified that a temperature of 13.8 °C is a critical temperature to limit the occurrence of *Ae. aegypti* [26]. A study into the occurrence of dengue fever was conducted in seven municipalities of Greater São Paulo in 2010–2013, showing that the months of January to May were those with the highest number of reported cases in the south-east of Brazil [32], explained by the authors as being a product of the higher rainfall in this area during those months. Our study in Campina Grande indicates that the infestation levels were higher during the months of May to September, which correlates with the higher levels of rainfall.

Regarding the socioeconomic context, a survey conducted in Rio de Janeiro observed that the locations of highest occurrence of classical dengue fever and haemorrhagic fever were not coincident, the latter being more prevalent in poor neighbourhoods and slums, providing clear evidence that social vulnerability may be a major factor influencing the occurrence of aggravation [16]. The present results corroborate these observations, demonstrating that the infestation and autocorrelation indices are most evident in contiguous neighbourhoods and the poorest ones.

To carry out the analysis of the LIRAa, neighbourhoods are grouped into strata with up to 12,000 inhabitants defined mostly by their sociodemographic, economic and physical characteristics. For this set of neighbourhoods, a single LIRAa value is obtained. This study found that the analysis of autocorrelation and spatial dependence between the neighbourhoods does not correspond to the demarcation of the strata. The autocorrelation analysis of mosquito infestation is more useful due to its ability to reveal patterns regarding risk analysis and vector management, including the definition of specific localities to be considered priority or a hotspot for the surveillance services.

The results of this work point to the need to revise the concept of stratum used as a unit to perform the LIRAa, considering that the surveillance services grouped together neighbourhoods that had different patterns of autocorrelation. The delimitation of stratum is based on the population density and some socioeconomic and geographical characteristics, parameters that should be revised considering the Modifiable Areal Unit Problem (MAUP) [33]. Spatial-based indicators, which use population density to define a territory, can produce biased results, considering that the summary values are influenced by both the shape and scale of the aggregation unit [33].

The outcomes of this study indicate that the inclusion of spatial and temporal autocorrelation analyses in the existing software (LIRAa System) used by the Brazilian Ministry of Health can provide a new strategy to identify the hotspots neighbourhoods, responsible for the most influence on mosquito infestation. This information is helpful for adopting and directing control vector actions with less impact on material and human resources. There is a gap in the literature in relation to the quantifiable associations between vector indices and dengue transmission that could reliably be used to predict outbreaks [15]. Further studies are required to elucidate the relationship between vector abundance and dengue transmission, which could be performed in comparable areas based on spatial autocorrelation methodologies. In the same municipality, it will be possible to compare the hotspot areas with high frequency of vector mosquitoes with those with low frequency to identify characteristics of each.

Regarding the limitations of this study, it should be considered that the analyses were carried out from secondary data provided by the Environmental Surveillance Service of Campina Grande municipality. In our analysis, environmental actions to curb infestations, as well as rainfall variability and climate change patterns, were not considered. For a better evaluation of the use of autocorrelation statistics to define strata or units for risk analysis and vector control, it will be important to replicate this study in other localities and assess the variability caused by environmental changes.

## Conclusions

To our knowledge, this study showed for the first time the autocorrelation patterns of *Ae. aegypti* infestation rates among neighbourhoods in the city of Campina Grande, in northeastern Brazil, using Moranʼs index, Moran mapping and LISA mapping. The use of spatial and temporal autocorrelation revealed hotpots or neighbourhoods that should be considered a priority to preventive actions of the entomological surveillance services. The predominance of high infestation rates and greater spatial dependence was observed between the months of May and July, the period with the highest rainfall in the city. This analysis is an innovative strategy capable of providing detailed information to the relevant public health authorities, which will enable a more efficient allocation of resources, particularly in mosquito prevention actions.

## Supplementary information

**Additional file 1: Figure S1.** Moranʼs index map for the house index (HI) showing the autocorrelation of *Ae. aegypti* mosquito infestation between neighbourhoods of Campina Grande city, Paraiba State, Brazil, in 2015.

**Additional file 2: Figure S2.** Moranʼs index map for the Breteau index (BI) showing the autocorrelation of *Ae. aegypti* mosquito infestation between neighbourhoods of Campina Grande city, Paraiba State, Brazil, in 2015.

**Additional file 3: Figure S3.** Moranʼs index map for the house index (HI) showing the autocorrelation of *Ae. aegypti* mosquito infestation between neighbourhoods of Campina Grande city, Paraiba State, Brazil, in 2016.

**Additional file 4: Figure S4.** Moranʼs index map for the Breteau index (BI) showing the autocorrelation of *Ae. aegypti* mosquito infestation between neighbourhoods of Campina Grande city, Paraiba State, Brazil, in 2016.

**Additional file 5: Figure S5.** Moran scatterplots of the HI data, the LISA maps, and the Moran maps in 2014.

**Additional file 6: Figure S6.** Moran scatterplots of the BI data, the LISA maps, and the Moran maps in 2014.

**Additional file 7: Figure S7.** Moran scatterplots of the HI data, the LISA maps, and the Moran maps in 2015.

**Additional file 8: Figure S8.** Moran scatterplots of the BI data, the LISA maps, and the Moran maps in 2015.

**Additional file 9: Figure S9.** Moran scatterplots of the HI data, the LISA maps, and the Moran maps in 2016.

**Additional file 10: Figure S10.** Moran scatterplots of the BI data, the LISA maps, and the Moran maps in 2016.

## Data Availability

Data supporting the conclusions of this article are included within the article and its additional files. The datasets used and/or analysed during the present study are available from the corresponding author upon reasonable request.

## Abbreviations

LIRAa: *Aedes aegypti* Rapid Index Survey
PNCD: Brazilian National Ministry of Dengue Control Programme
HI: house index
BI: Breteau index
ACEs: endemic disease control agents
