# Supplementary figures and images for: Spatial modelling of the infestation indices of Aedes aegypti: an innovative strategy for vector control actions in developing countries
Source: Parasit Vectors. 2020 Apr 16;13:197. doi: 10.1186/s13071-020-04070-w (PMC7164210; doi:10.1186/s13071-020-04070-w)

# Map of Quartiles HI - 2015

LIRAA 1 - January

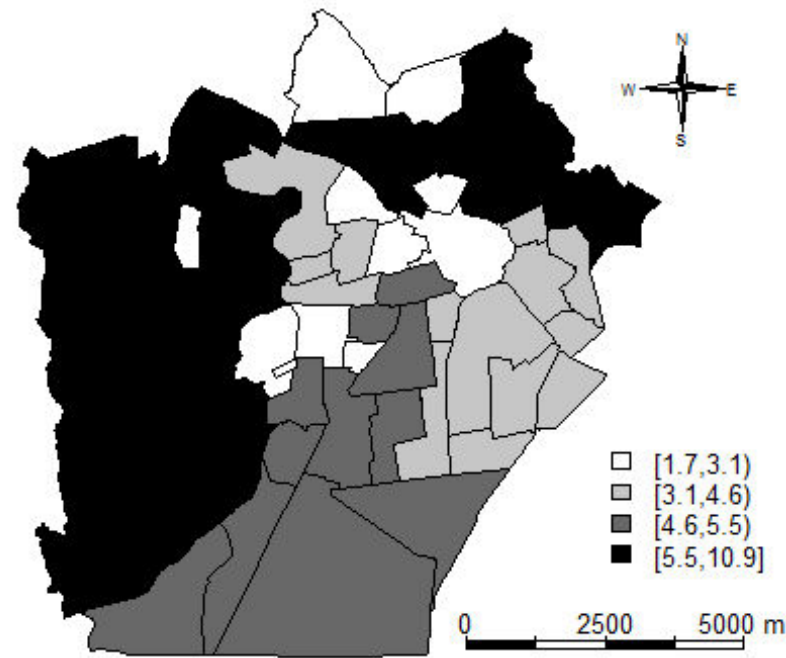

LIRAA 2 - March

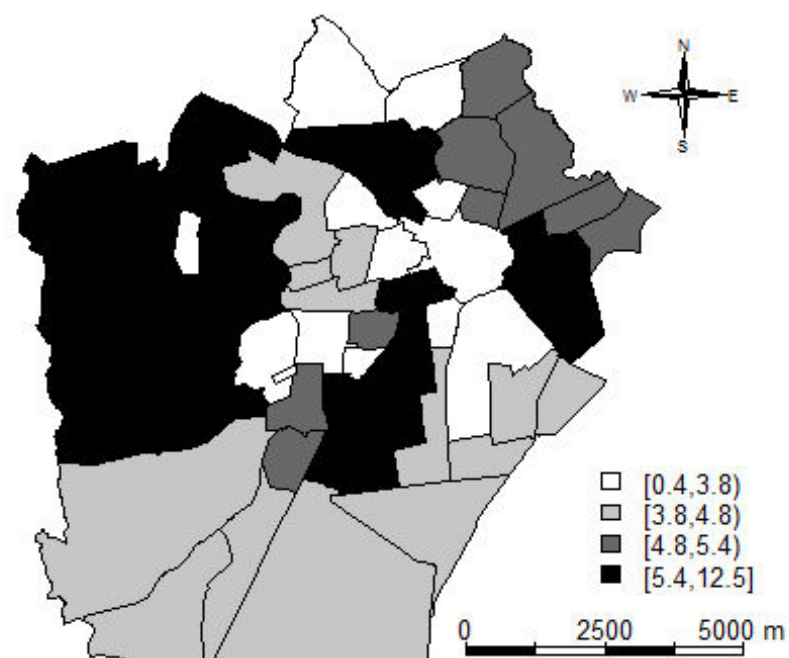

LIRAA 3 - July

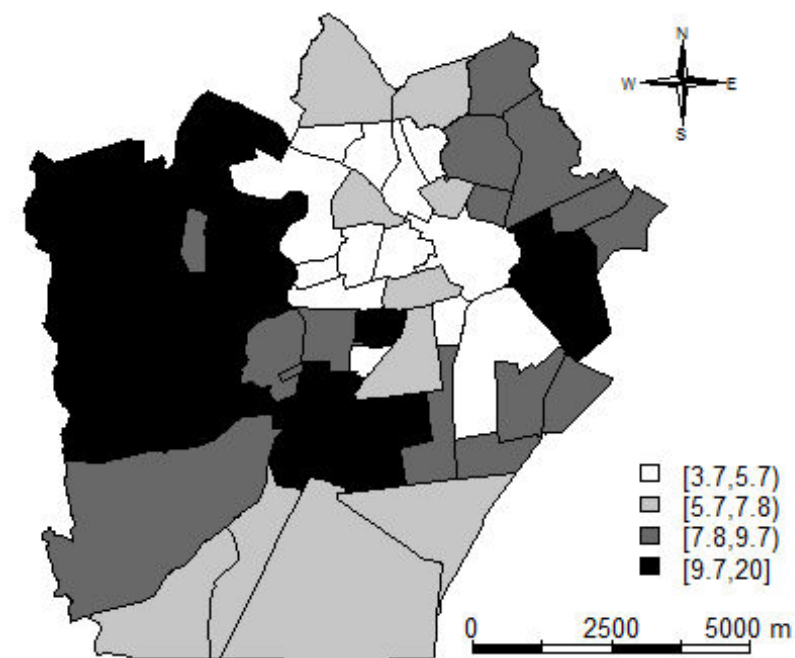

LIRAA 4 - October

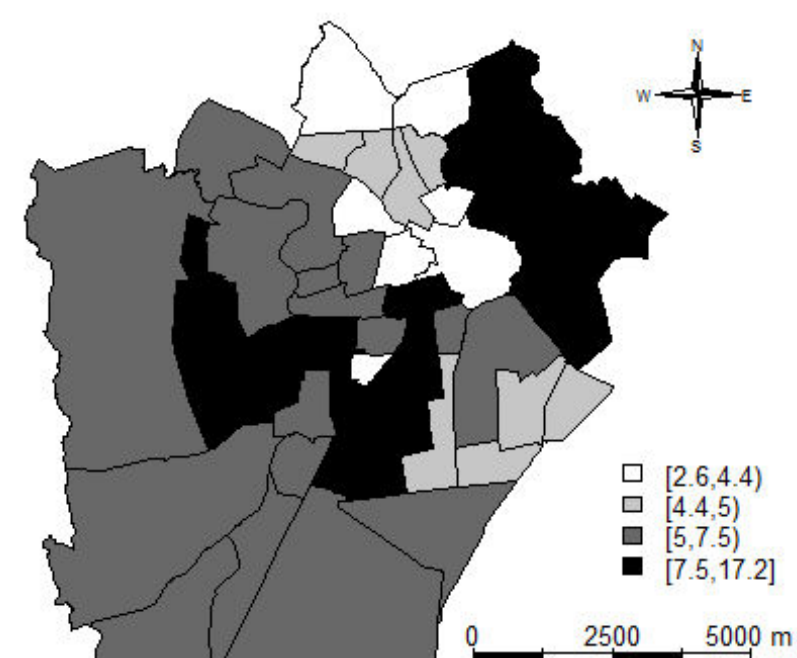

Supplement: Supplementary file 1 — Additional file 1: Figure S1. Moranʼs index map for the house index (HI) showing the autocorrelation of Ae. aegypti mosquito infestation between neighbourhoods of Campina Grande city, Paraiba State, Brazil, in 2015. [file 13071_2020_4070_MOESM1_ESM.pdf]

# Map of Quartiles BI - 2015

LIRaA 1 - January

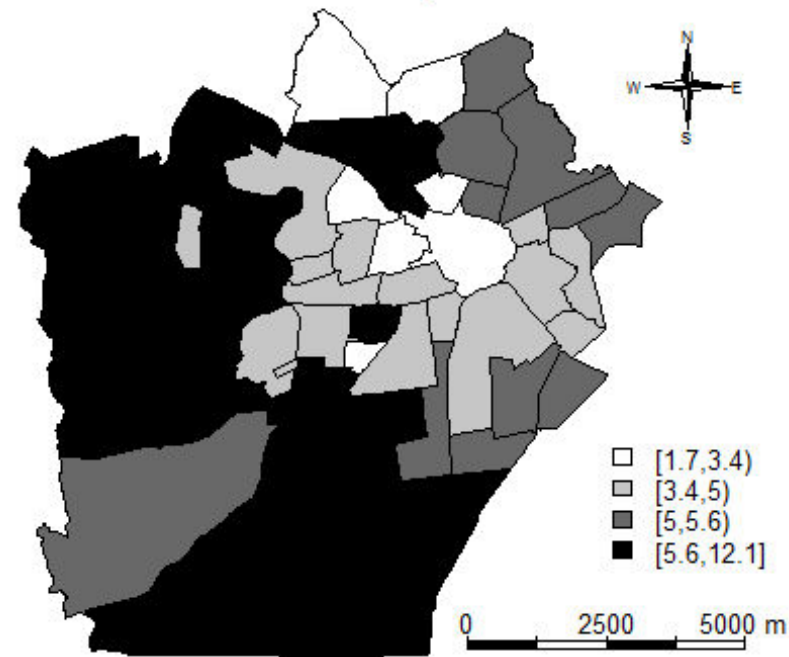

LIRaA 2 - March

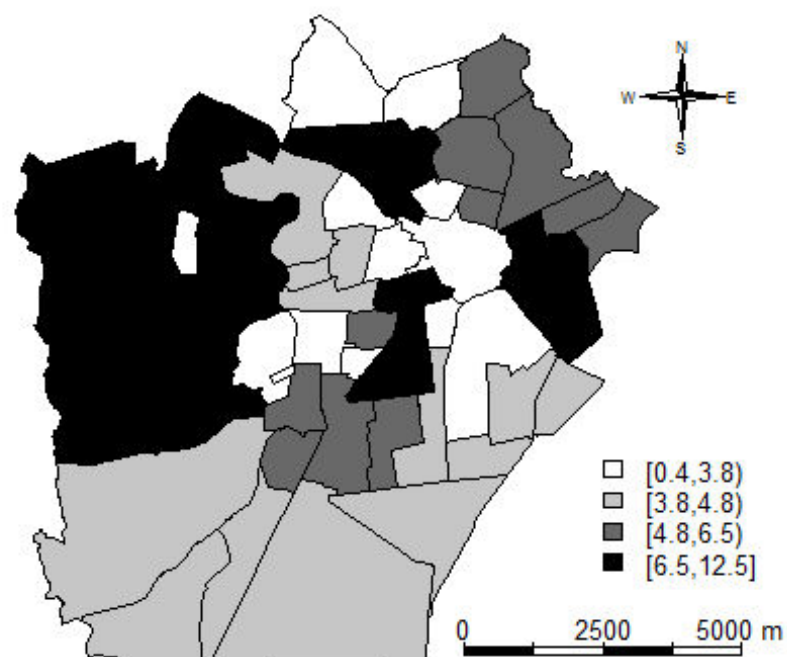

LIRaA 3 - July

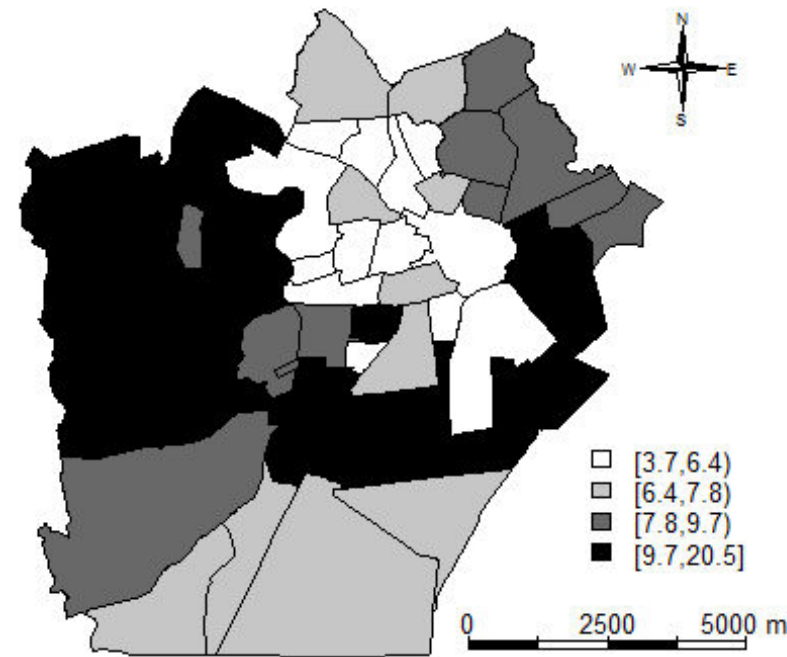

LIRaA 4 - October

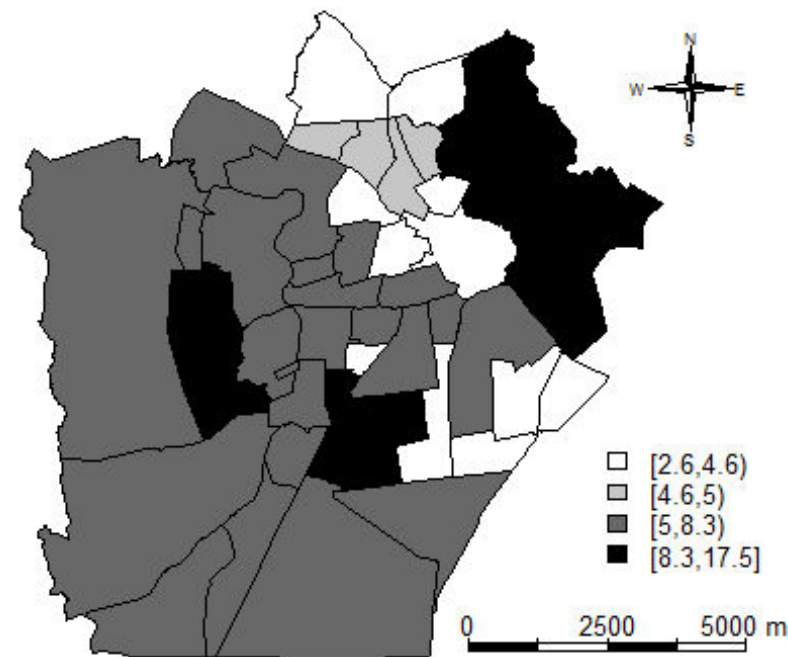

Supplement: Supplementary file 2 — Additional file 2: Figure S2. Moranʼs index map for the Breteau index (BI) showing the autocorrelation of Ae. aegypti mosquito infestation between neighbourhoods of Campina Grande city, Paraiba State, Brazil, in 2015. [file 13071_2020_4070_MOESM2_ESM.pdf]

## Map of Quartiles HI - 2016

LIRAA 1 - April

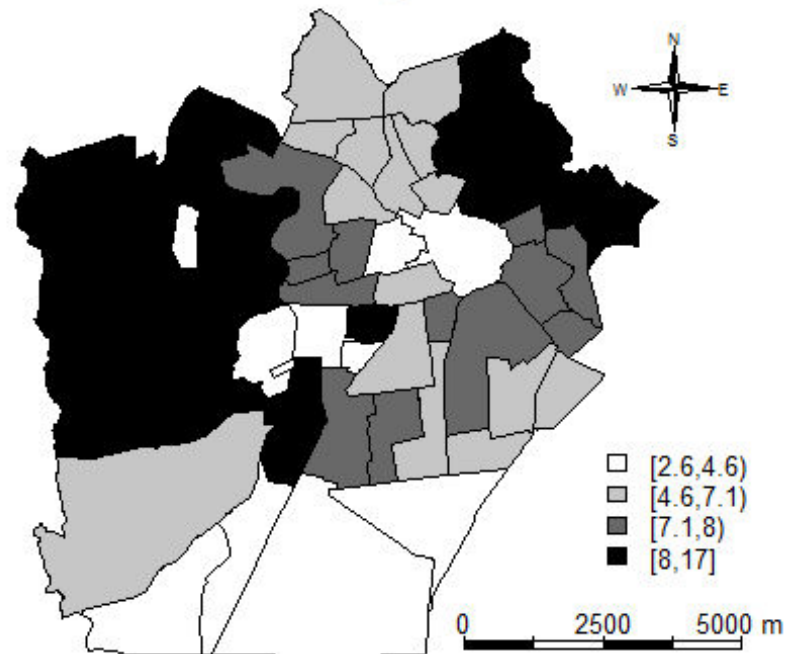

LIRAA 2 - July

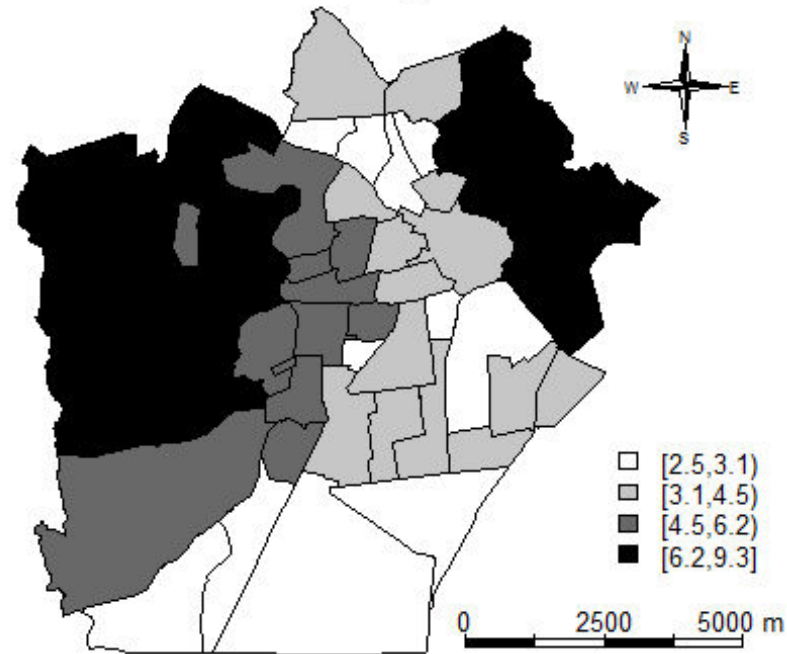

LIRAA 3 - October

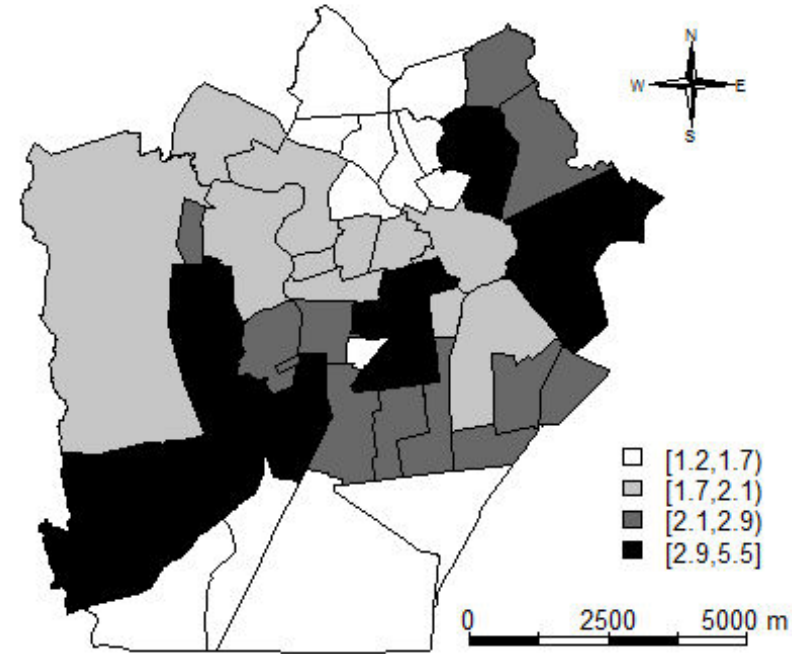

Supplement: Supplementary file 3 — Additional file 3: Figure S3. Moranʼs index map for the house index (HI) showing the autocorrelation of Ae. aegypti mosquito infestation between neighbourhoods of Campina Grande city, Paraiba State, Brazil, in 2016. [file 13071_2020_4070_MOESM3_ESM.pdf]

# Map of Quartiles BI - 2016

LIRaA 1 - April

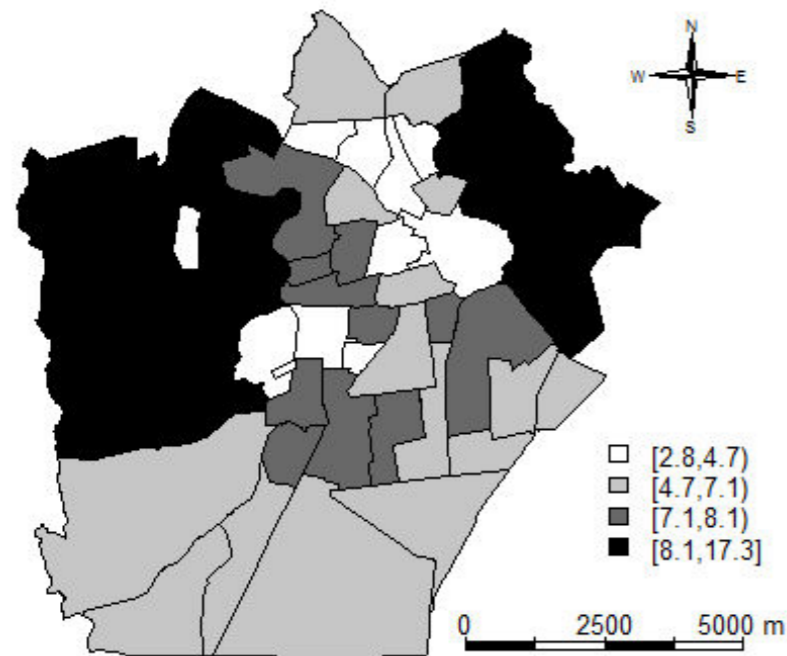

LIRaA 2 - July

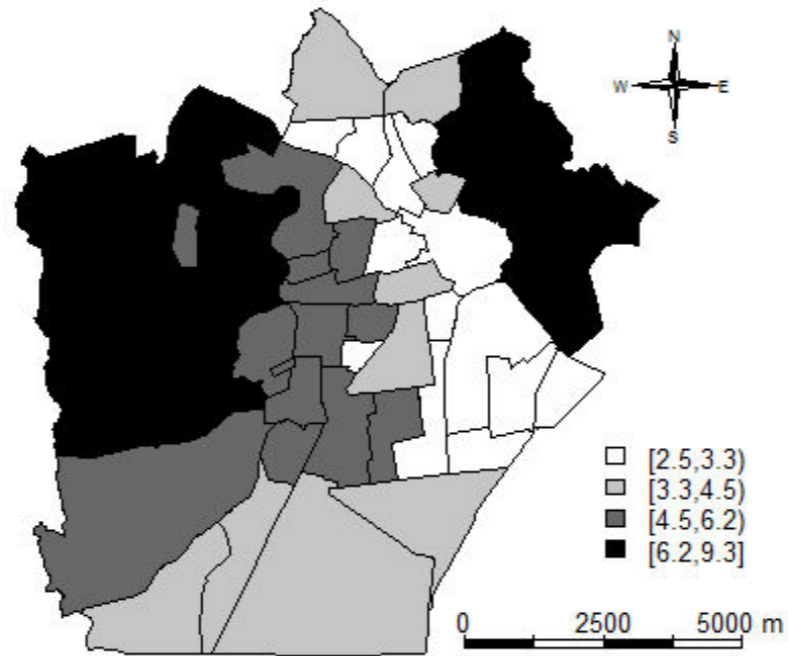

LIRaA 3 - October

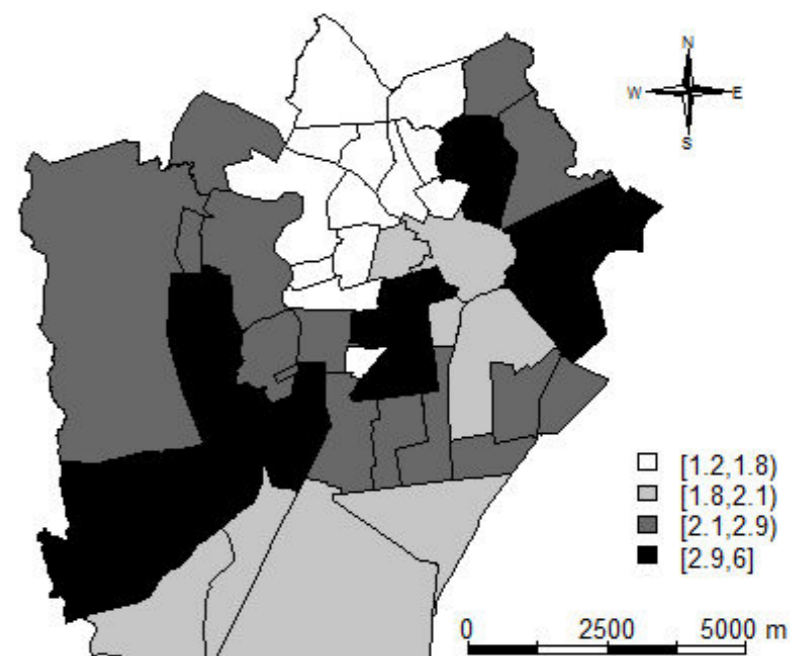

Supplement: Supplementary file 4 — Additional file 4: Figure S4. Moranʼs index map for the Breteau index (BI) showing the autocorrelation of Ae. aegypti mosquito infestation between neighbourhoods of Campina Grande city, Paraiba State, Brazil, in 2016. [file 13071_2020_4070_MOESM4_ESM.pdf]

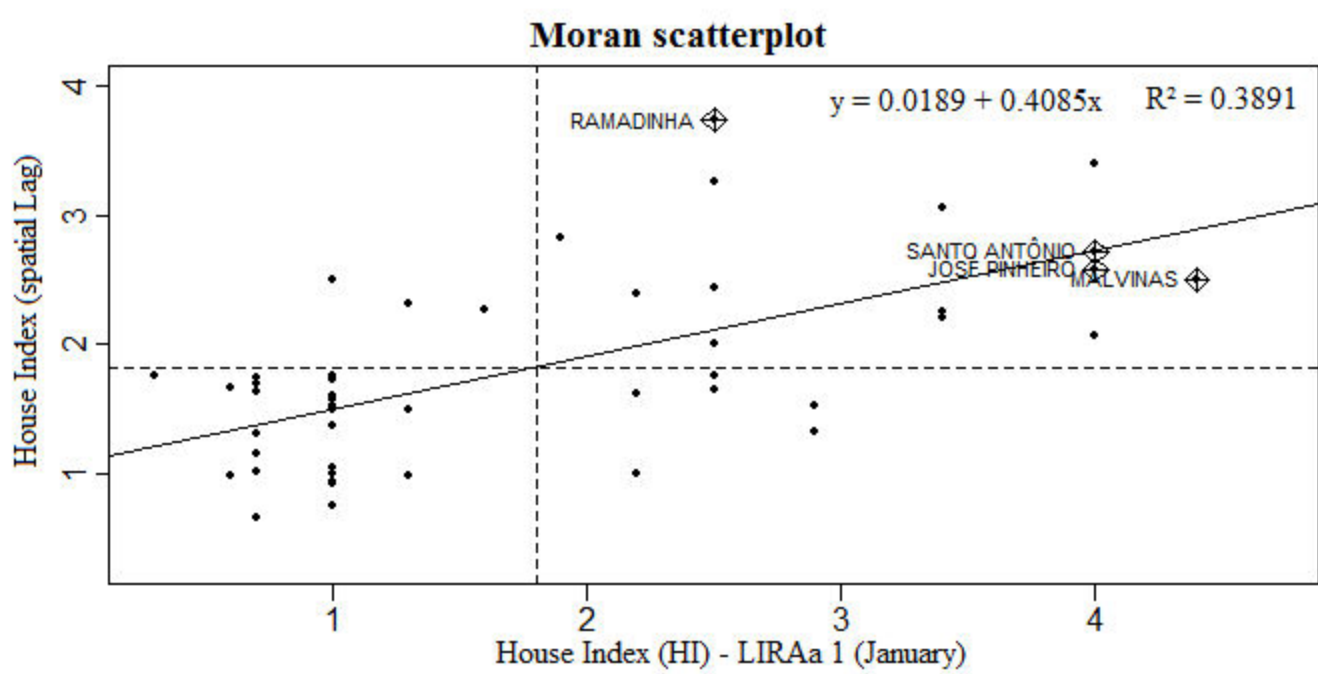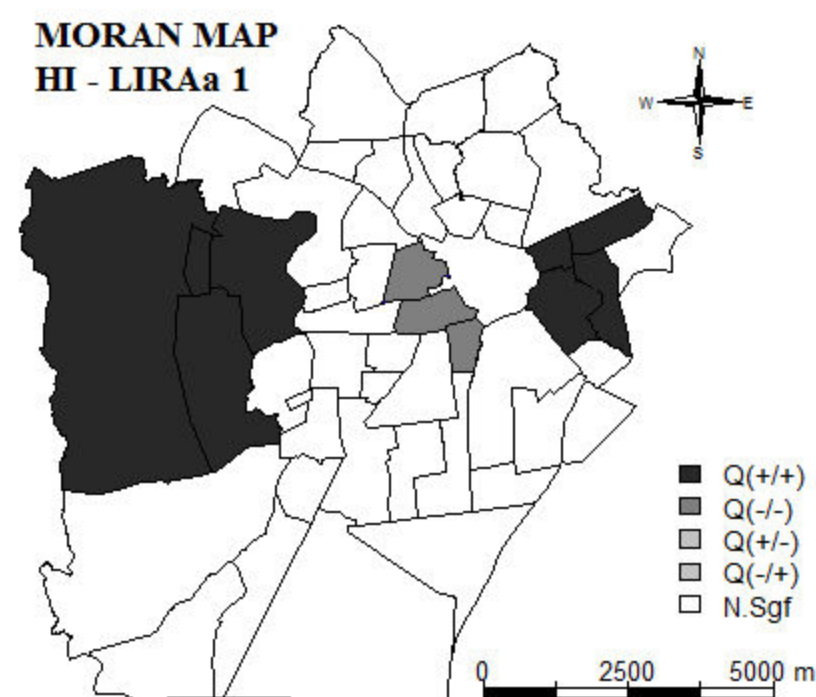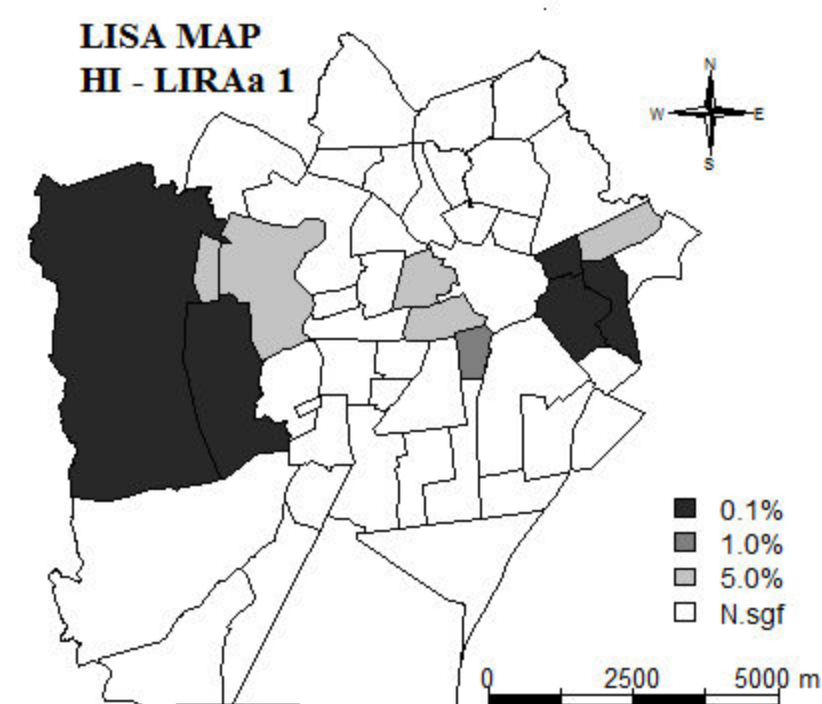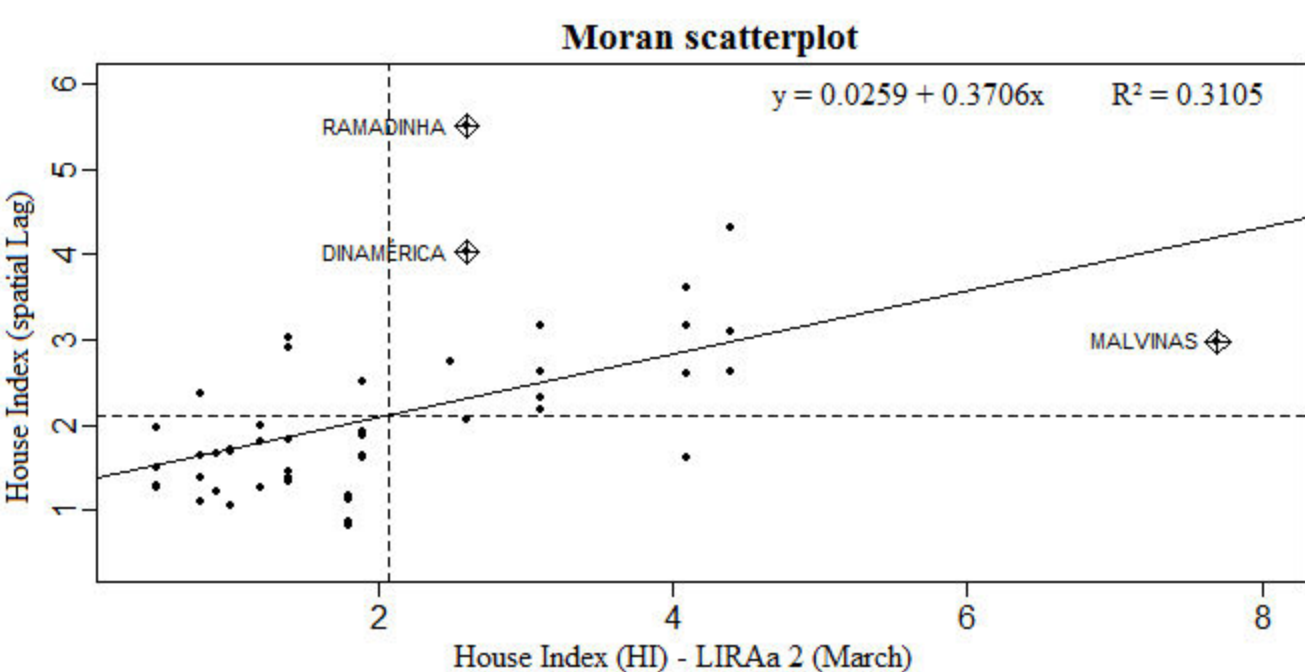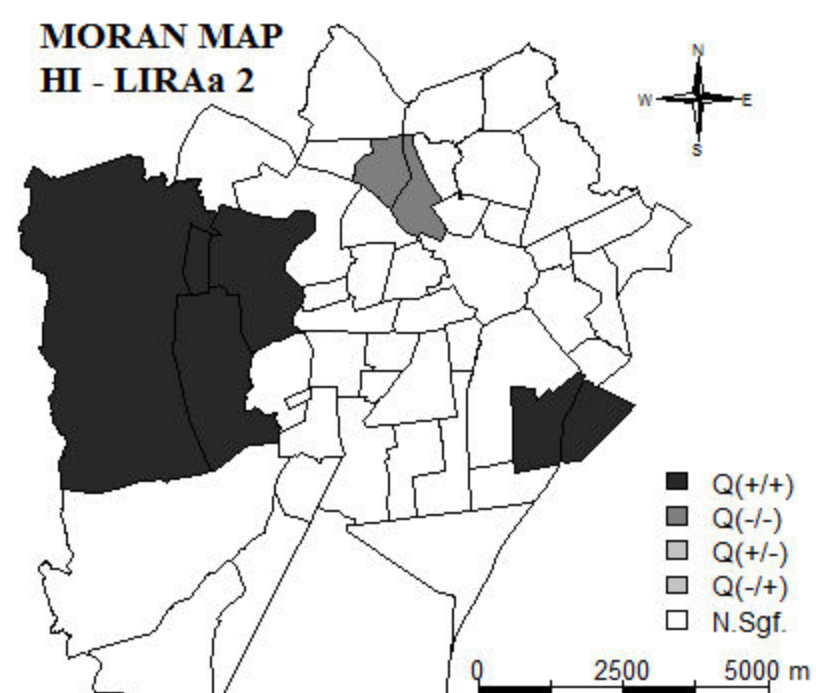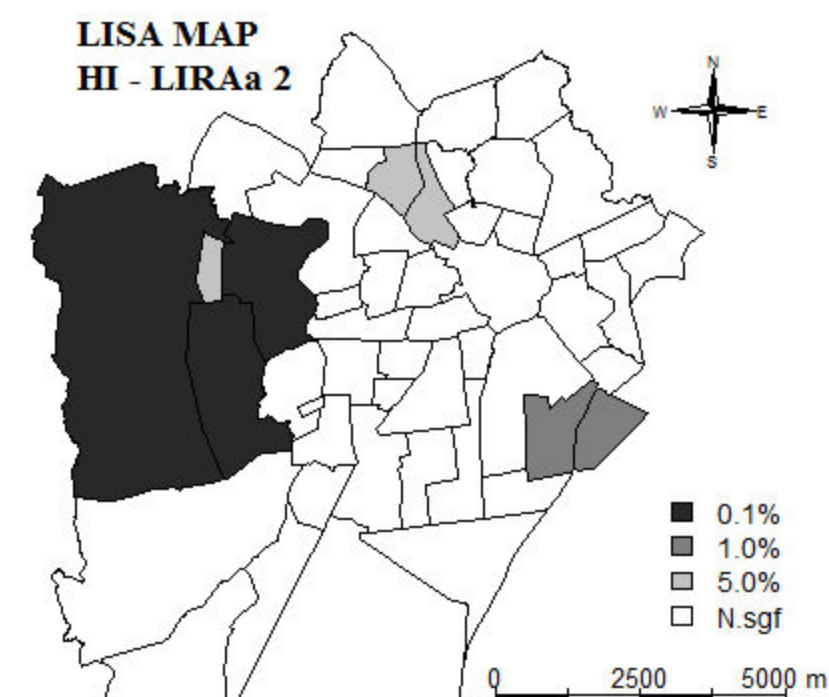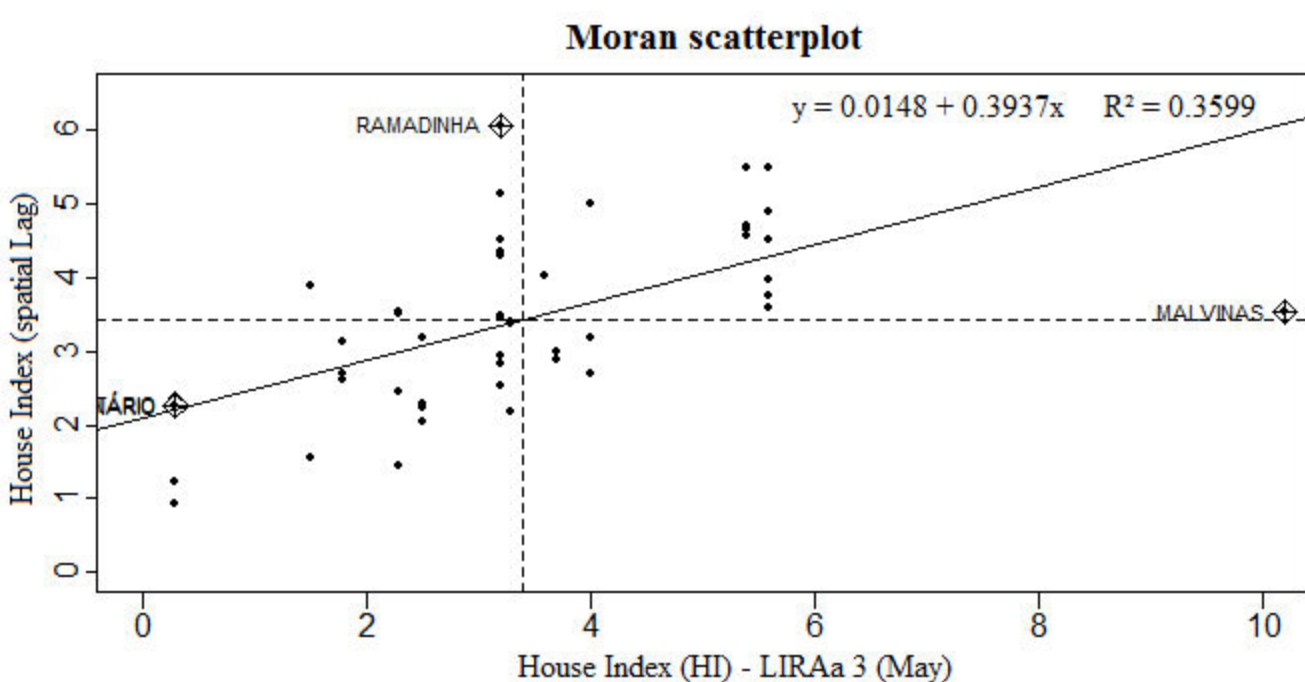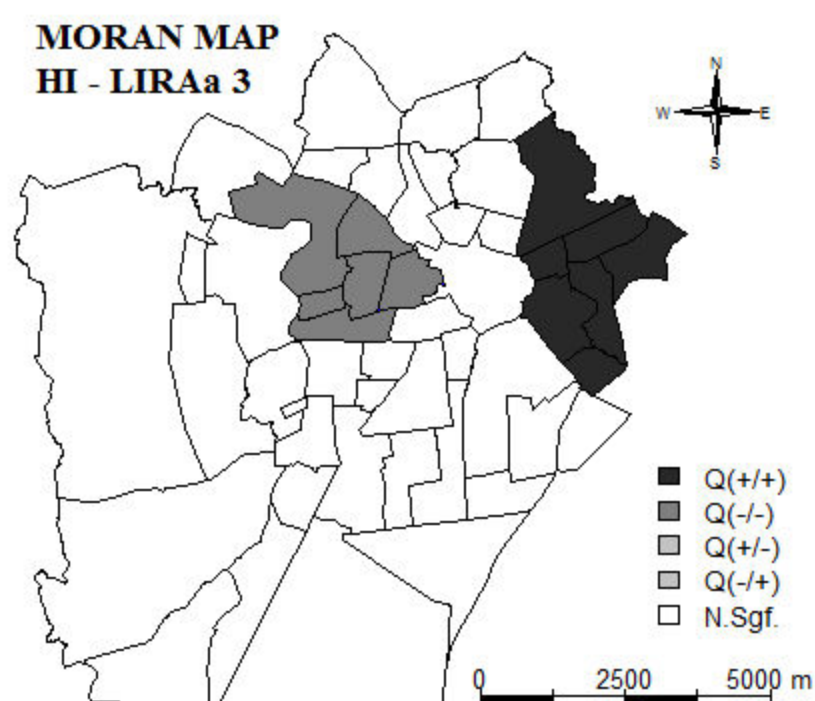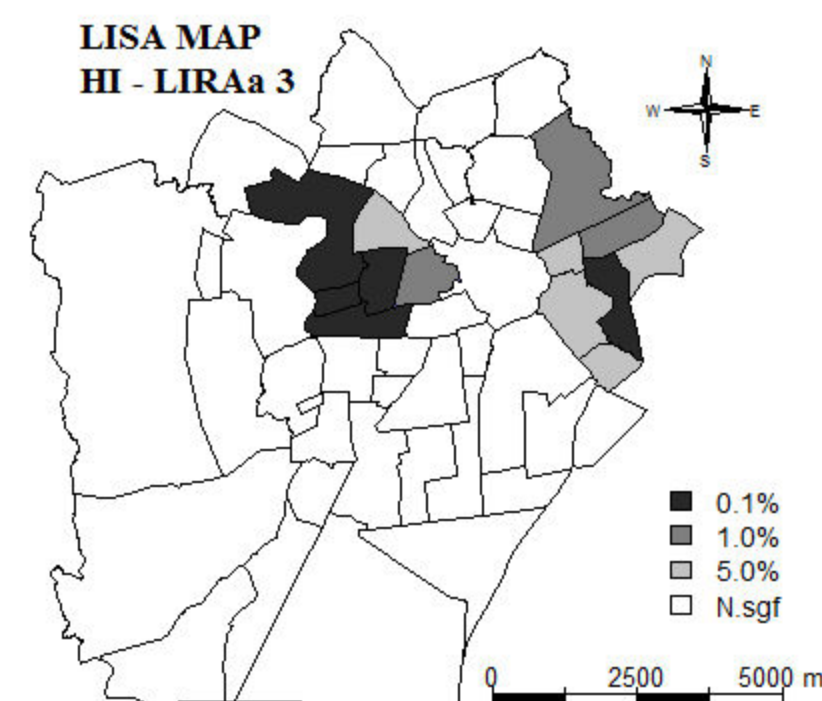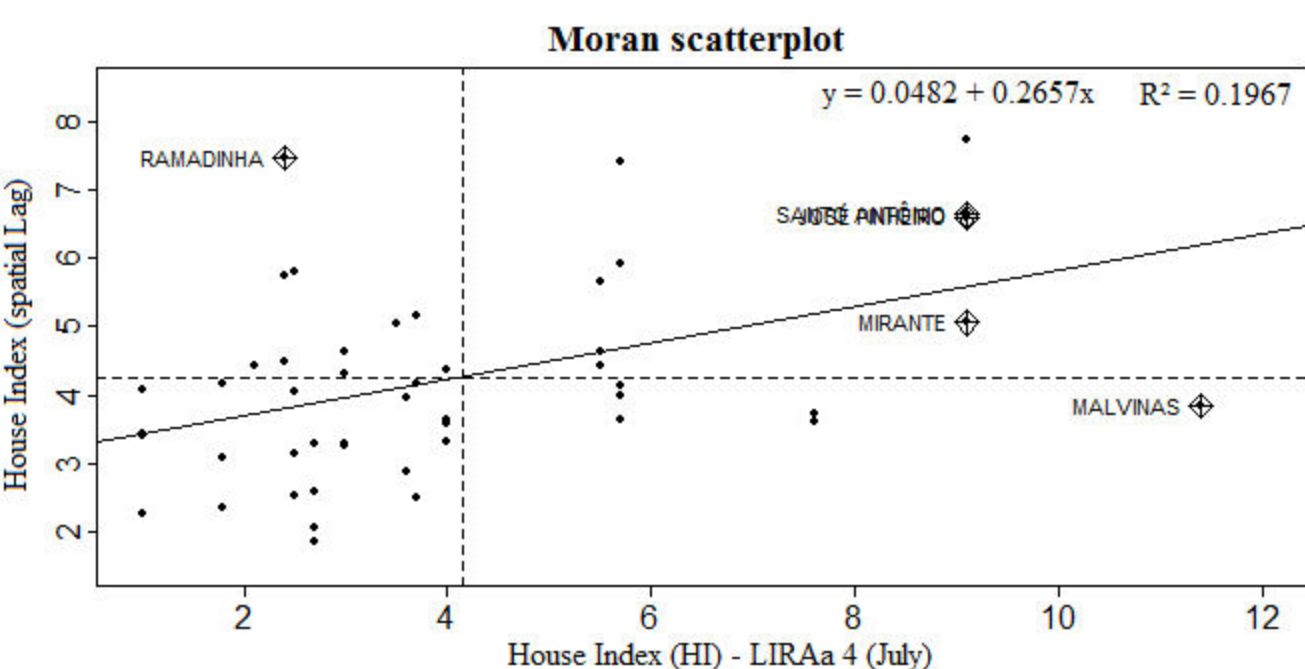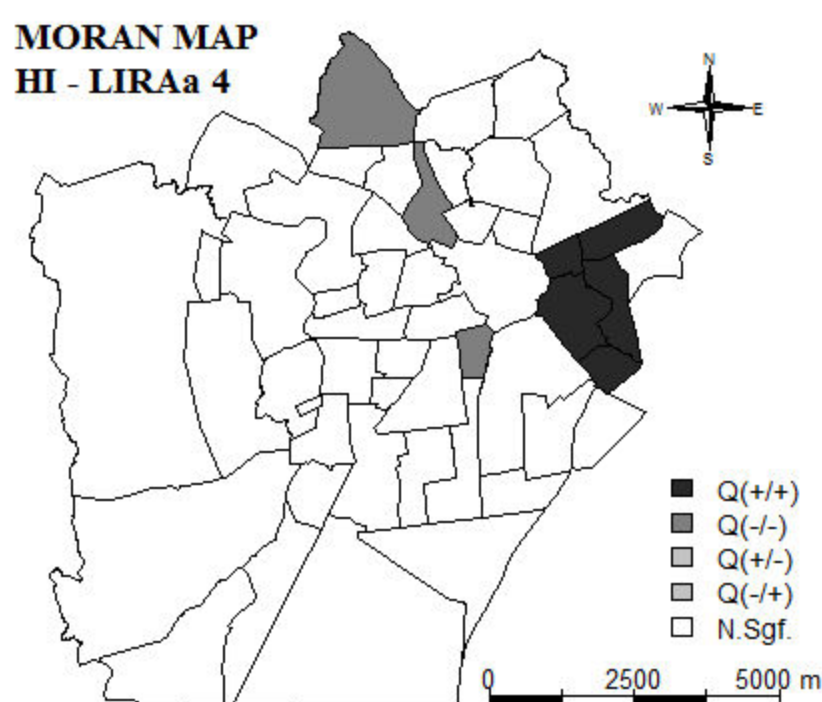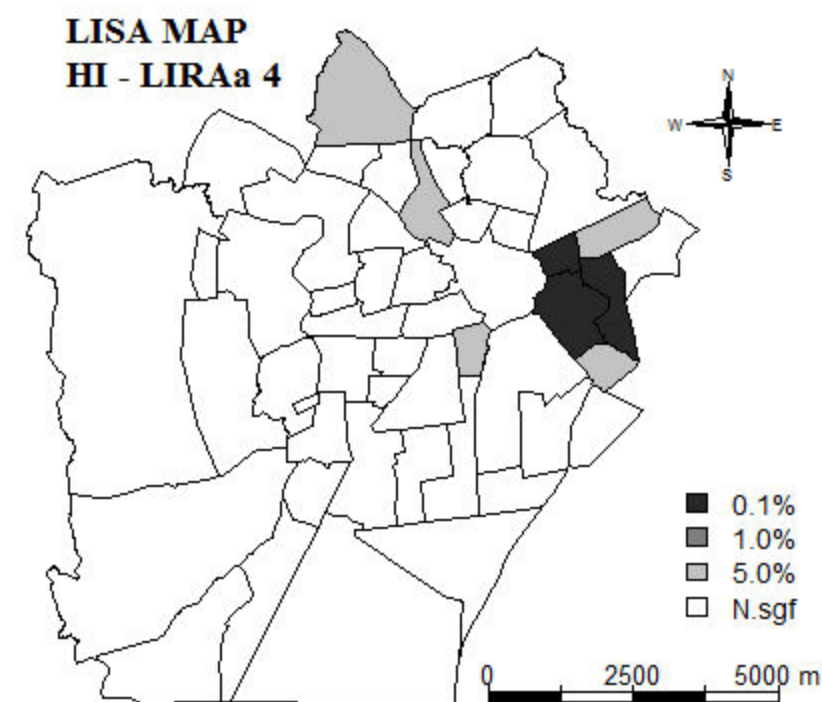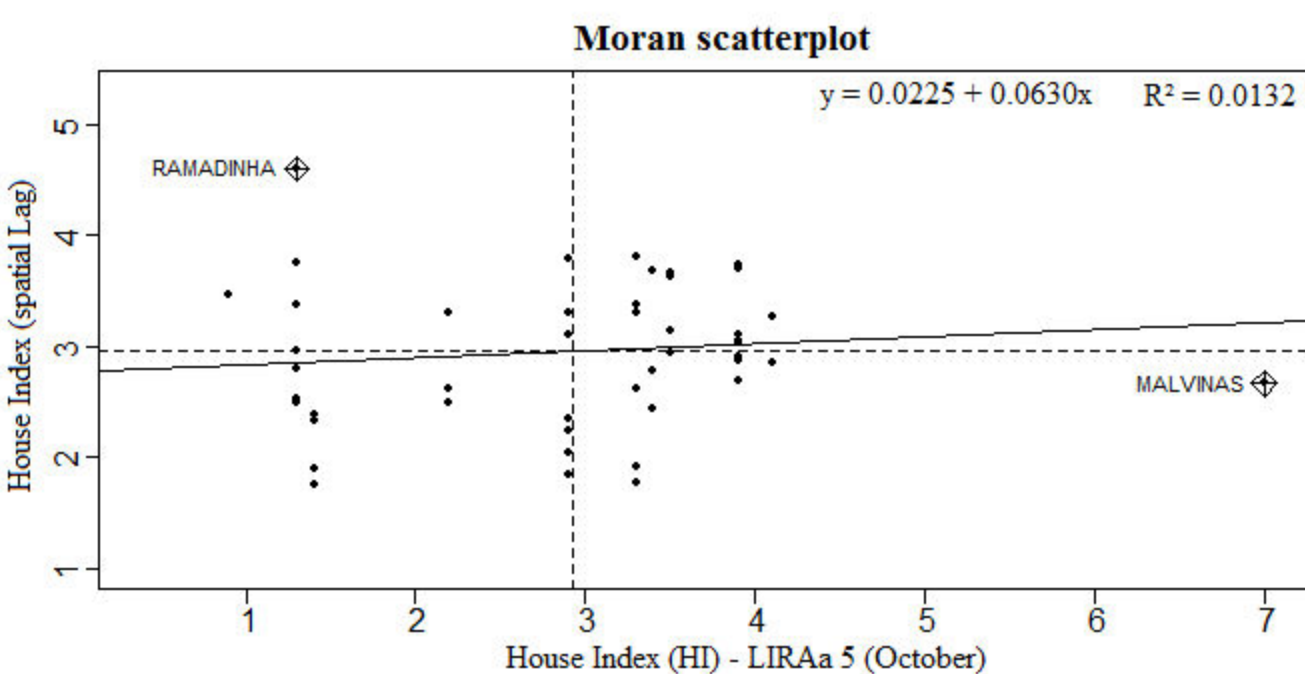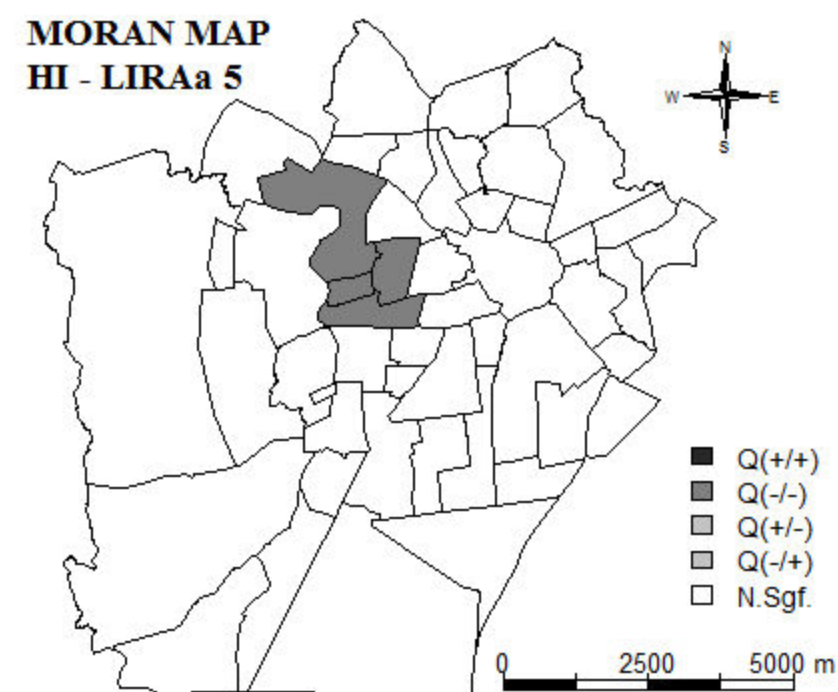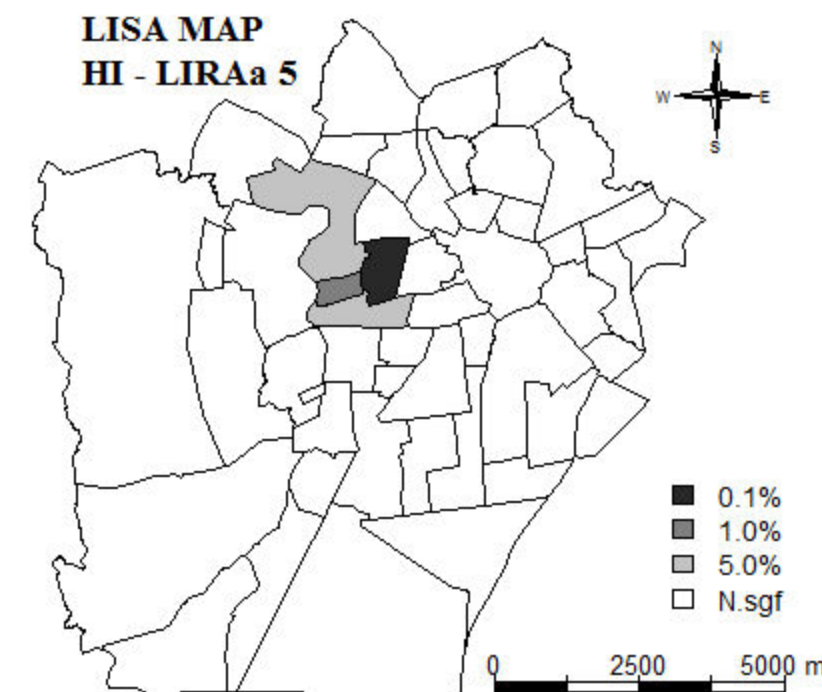

Supplement: Supplementary file 5 — Additional file 5: Figure S5. Moran scatterplots of the HI data, the LISA maps, and the Moran maps in 2014. [file 13071_2020_4070_MOESM5_ESM.pdf]

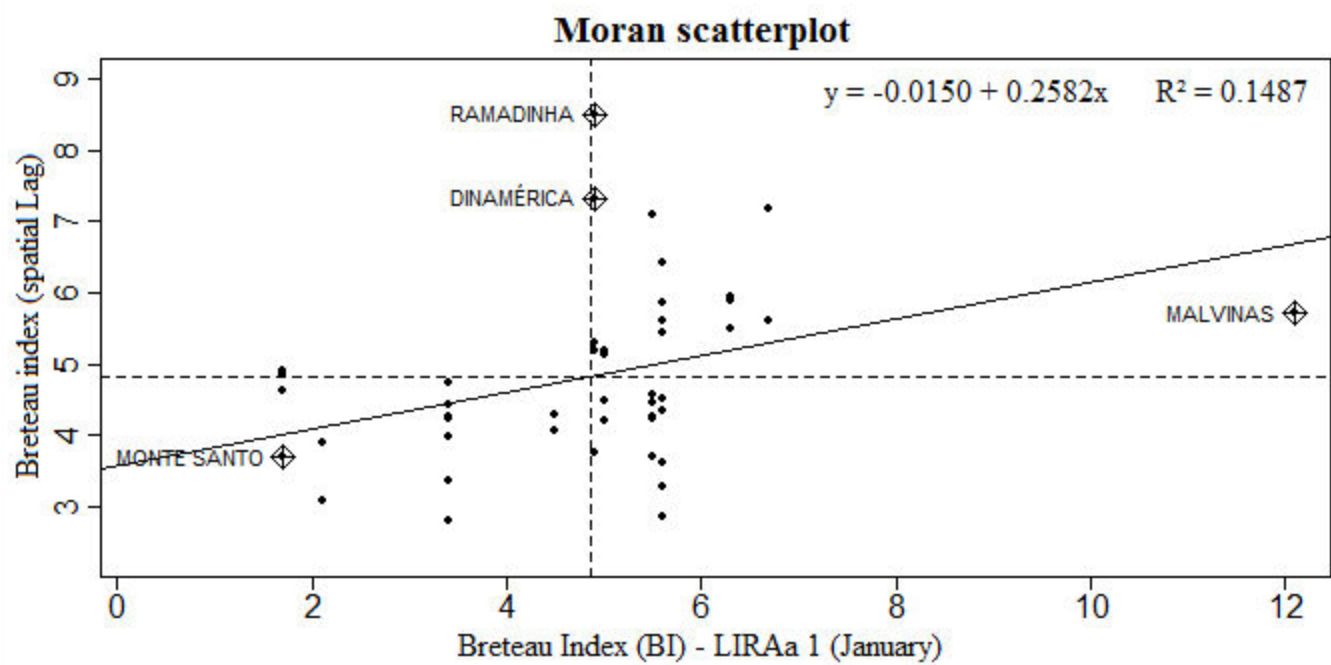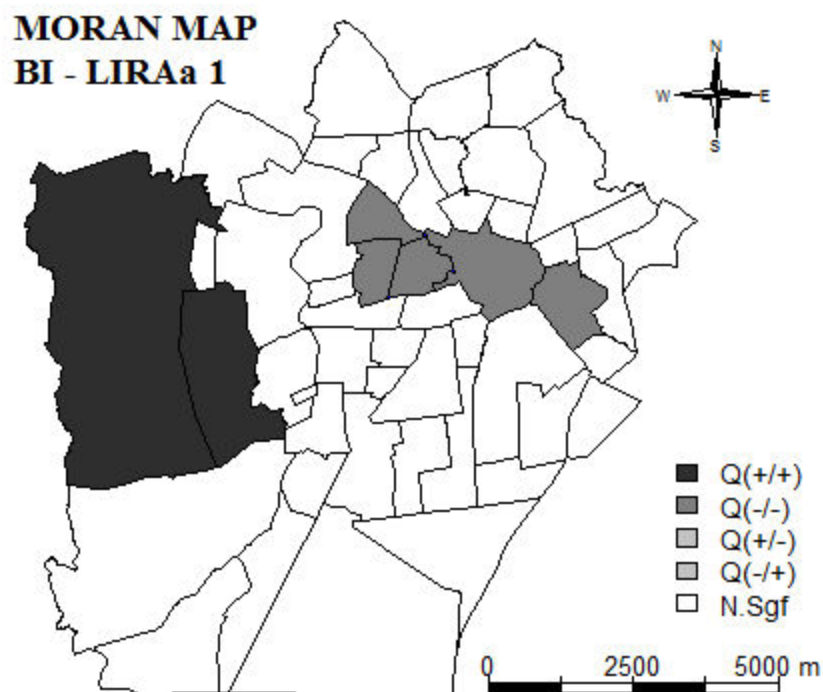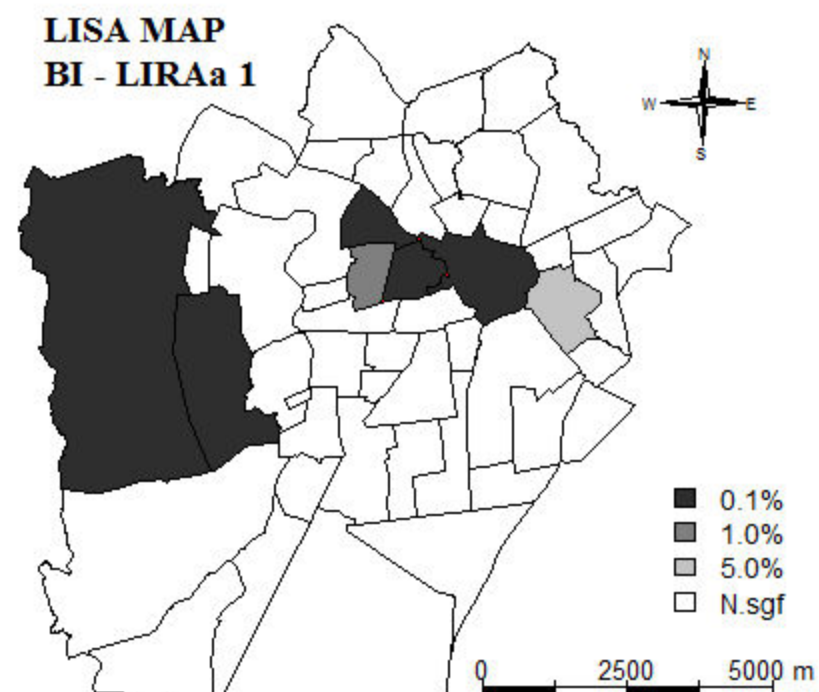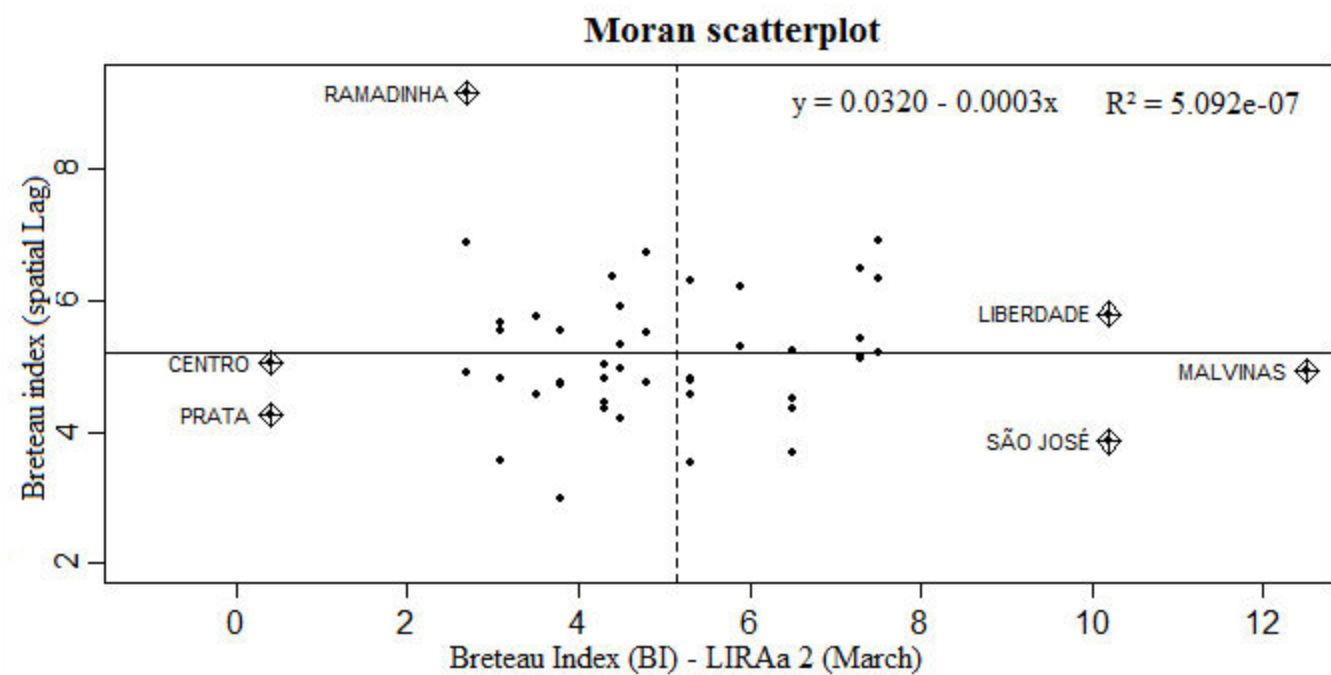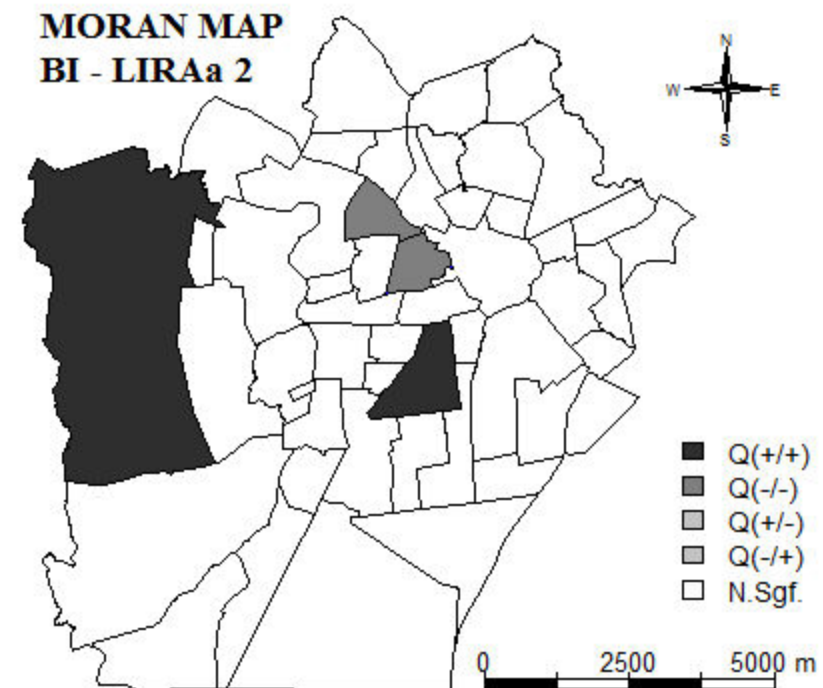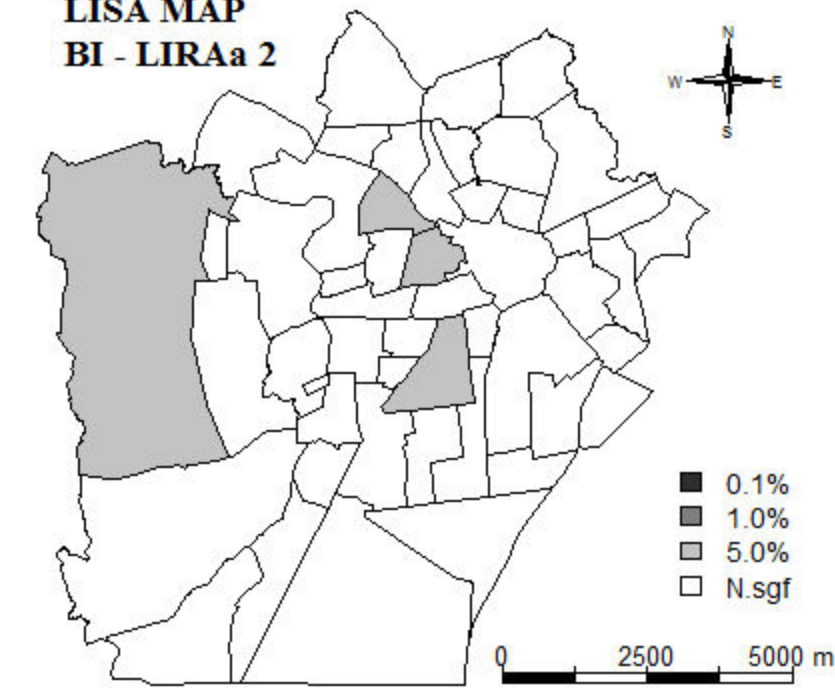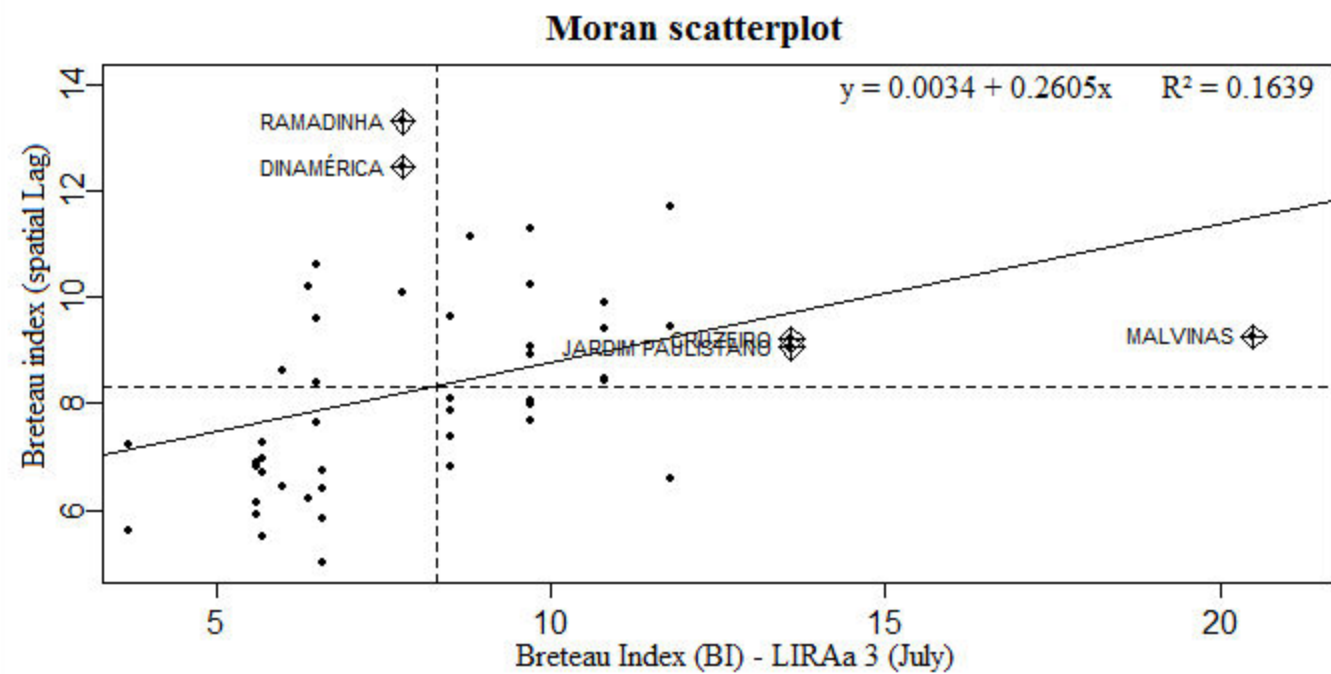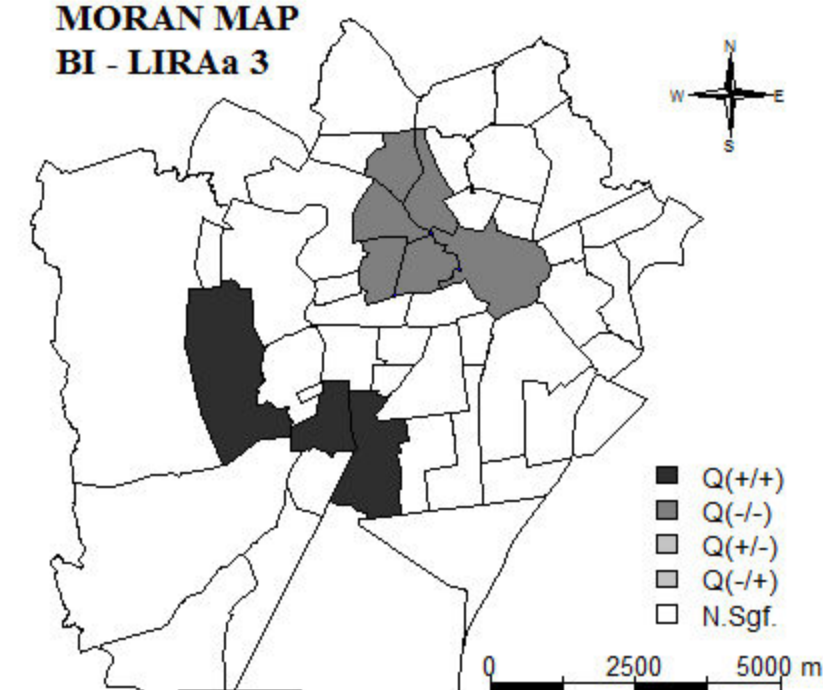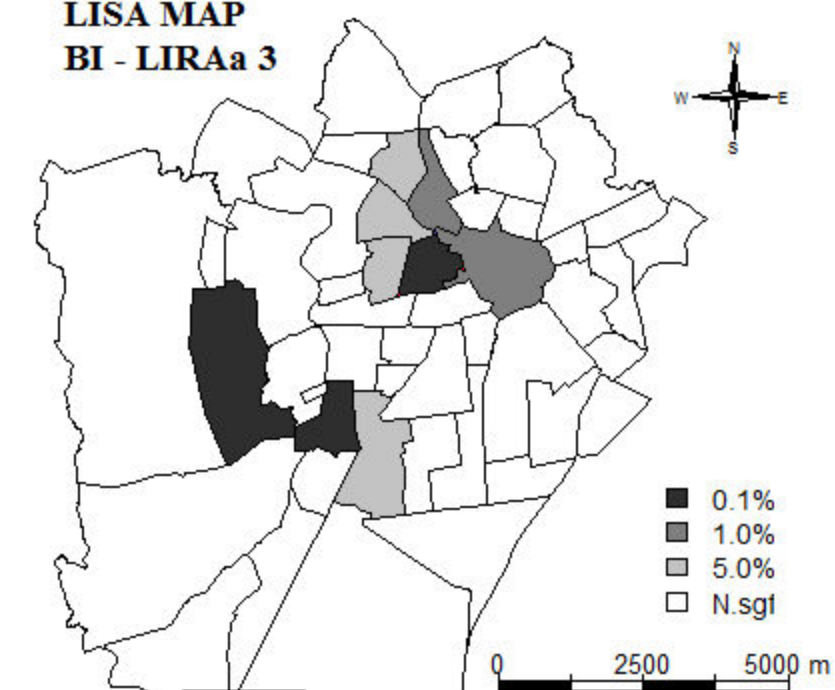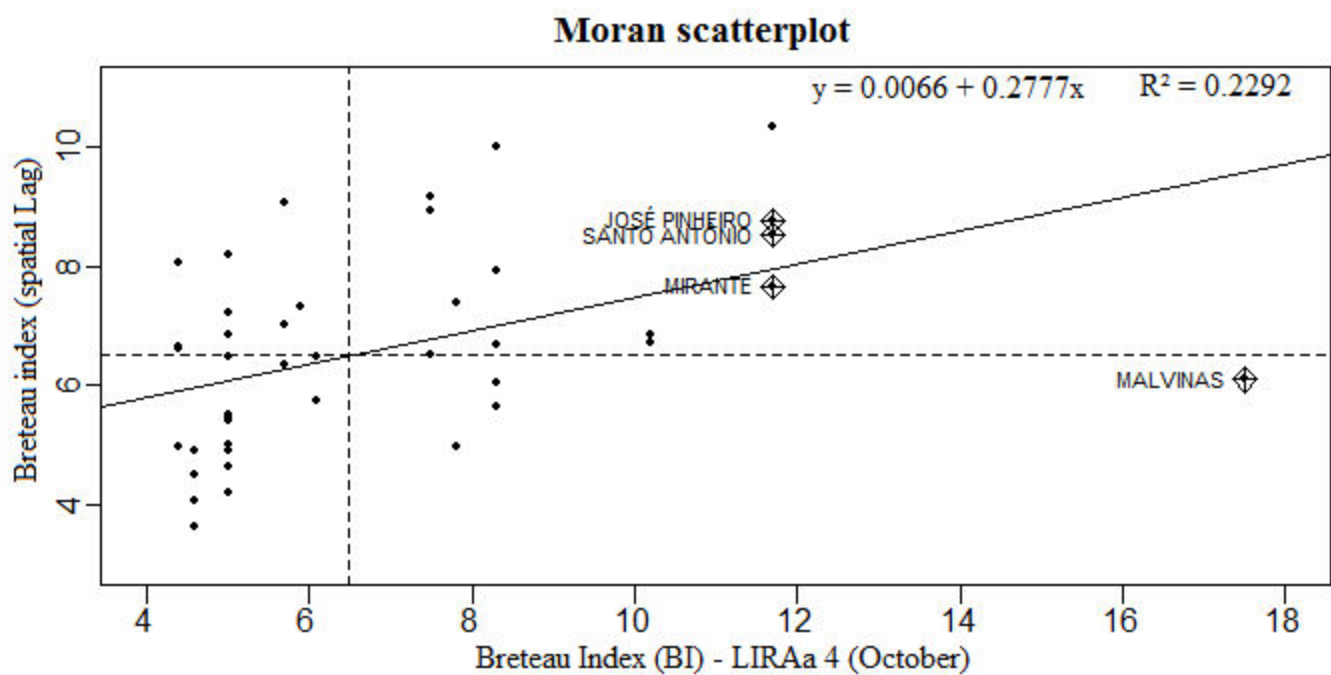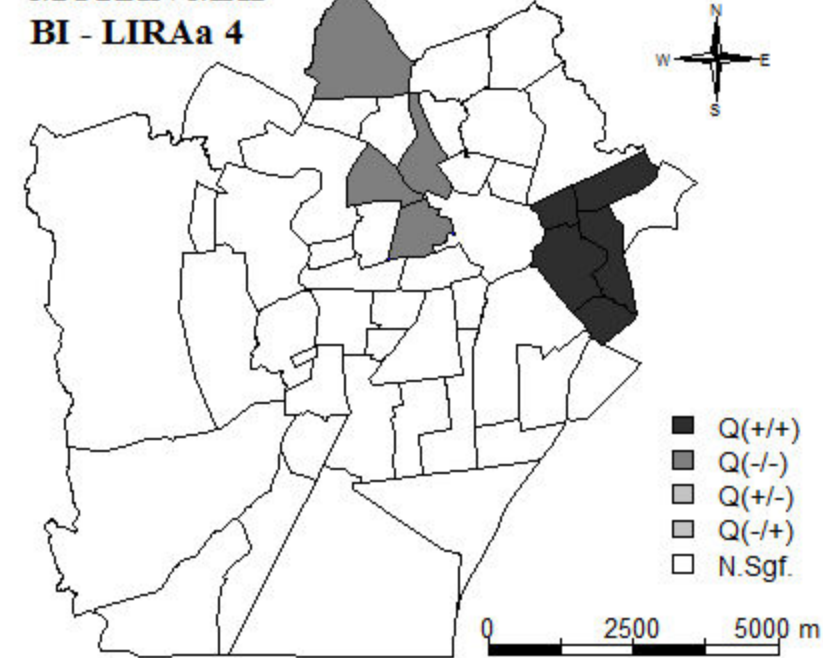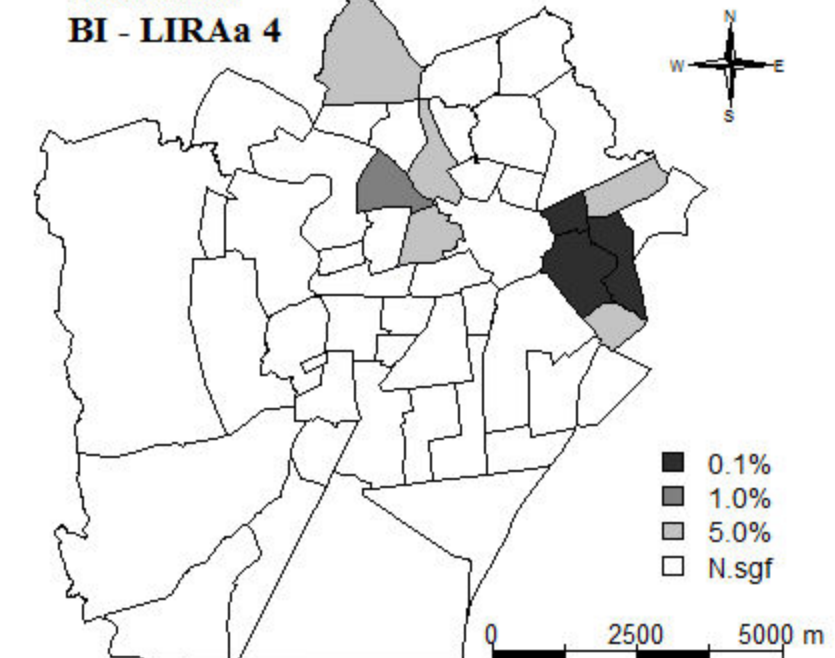

Supplement: Supplementary file 8 — Additional file 8: Figure S8. Moran scatterplots of the BI data, the LISA maps, and the Moran maps in 2015. [file 13071_2020_4070_MOESM8_ESM.pdf]

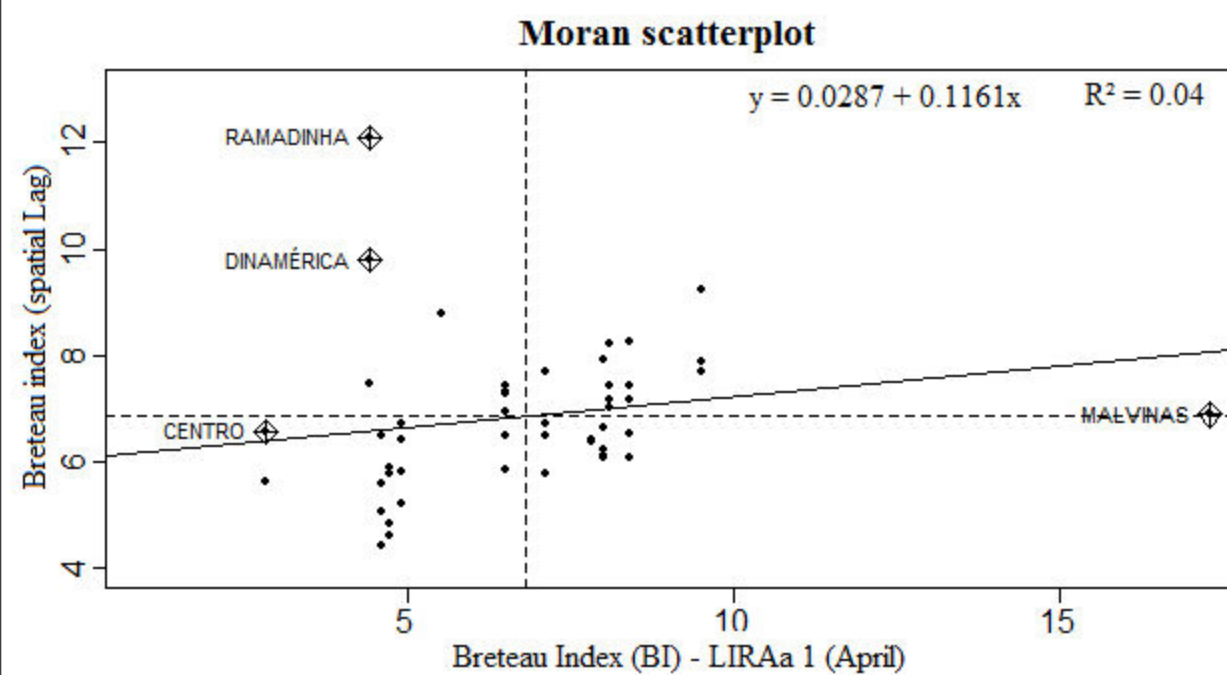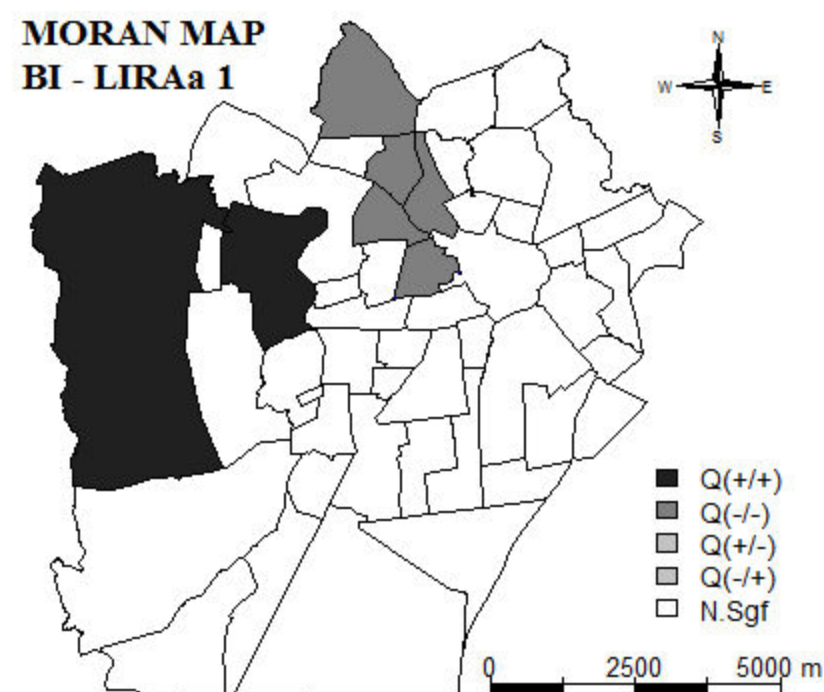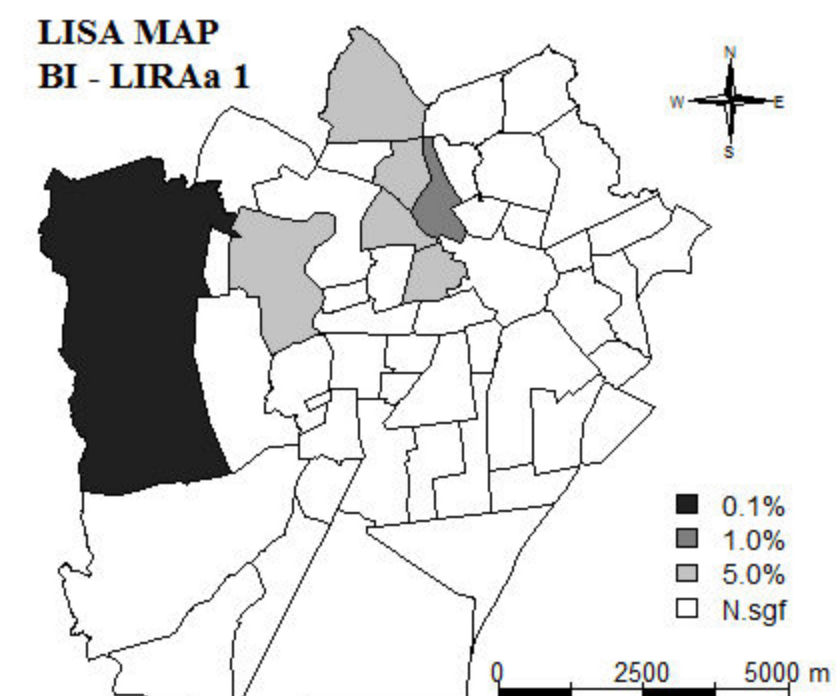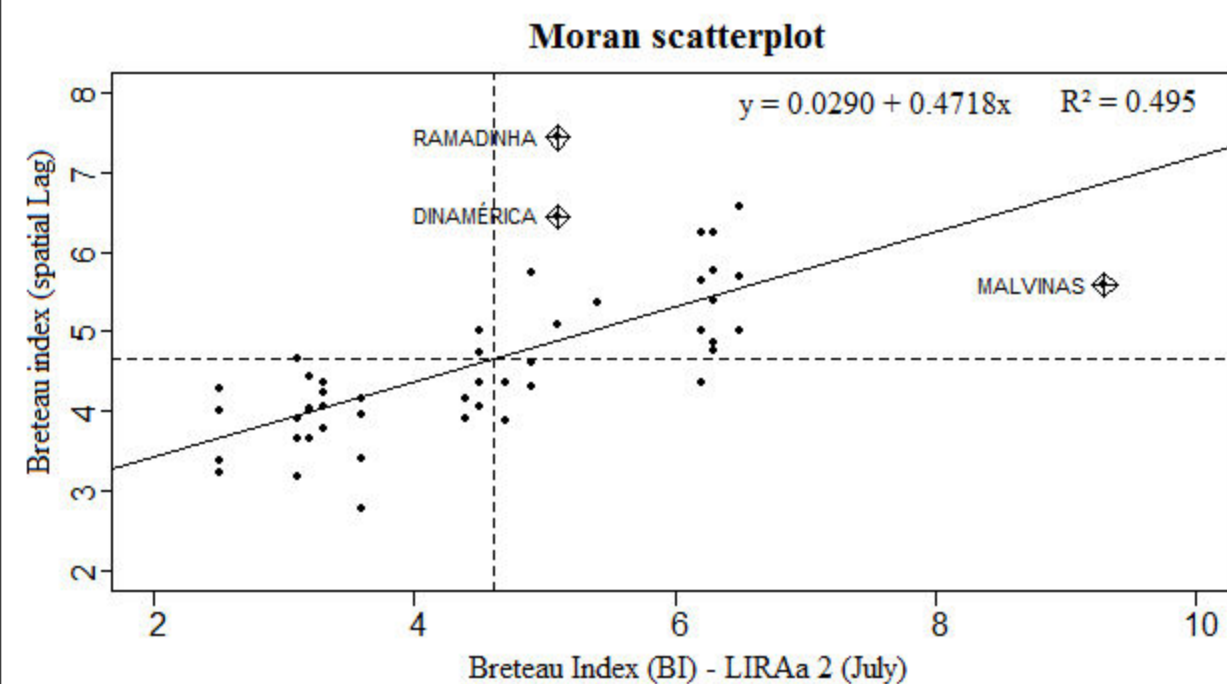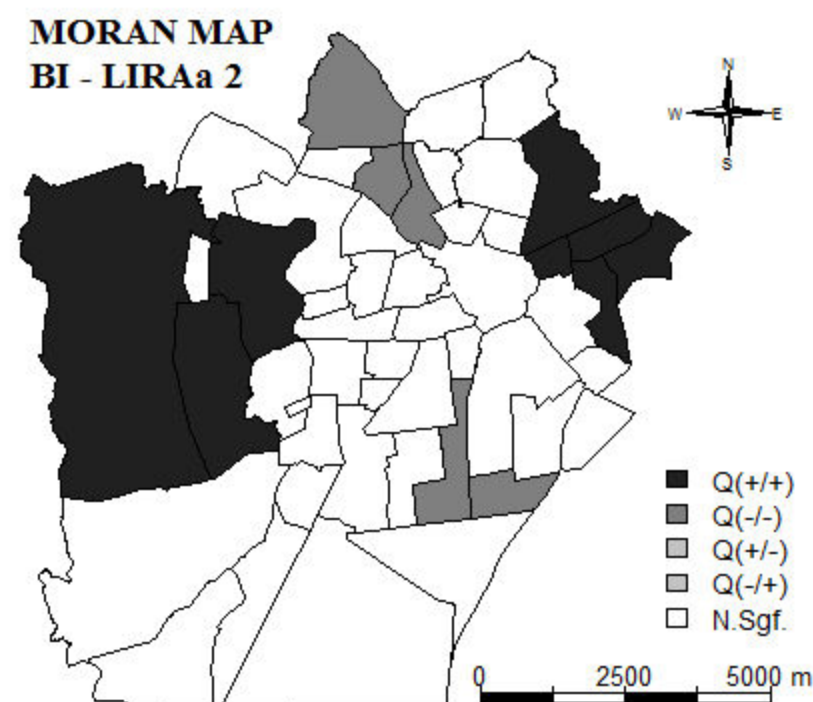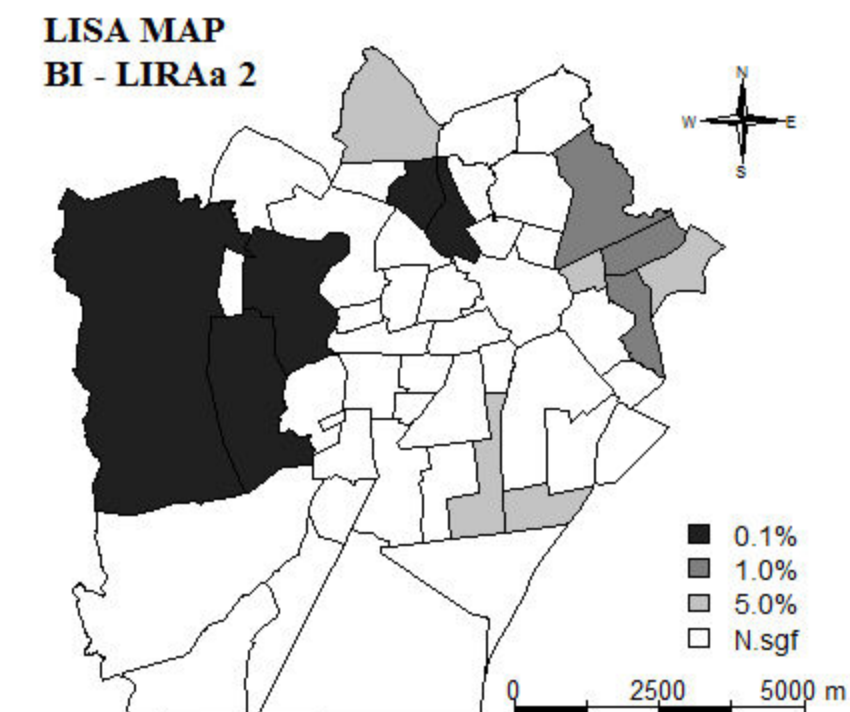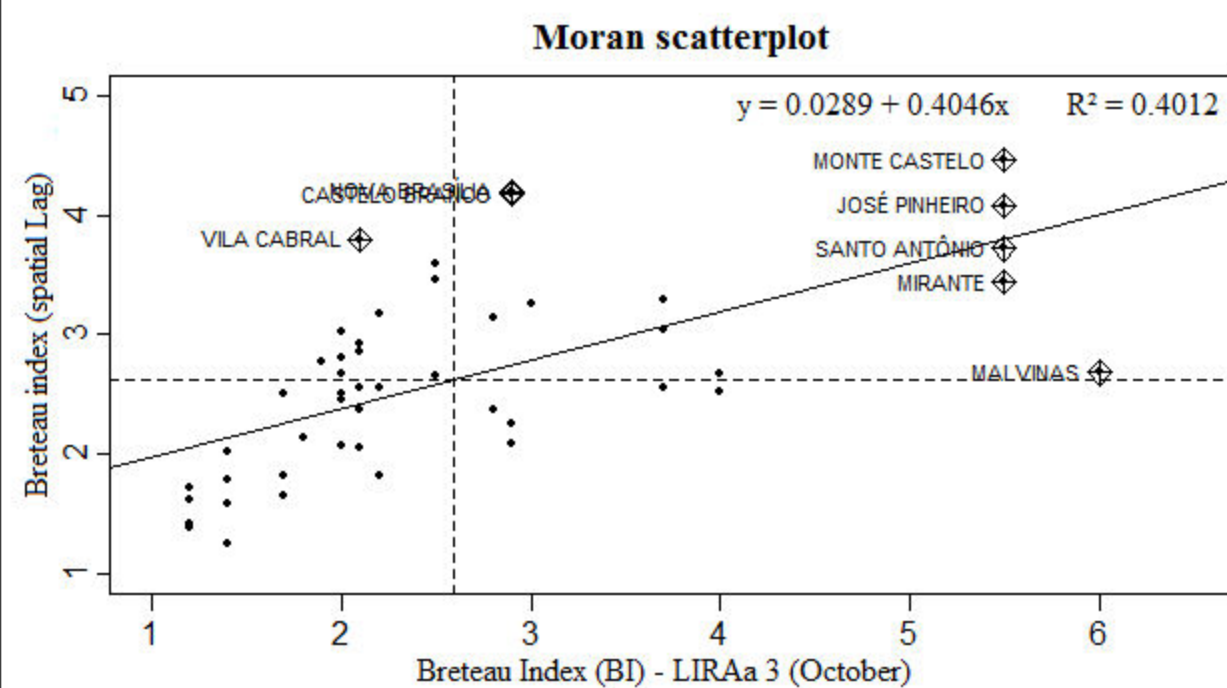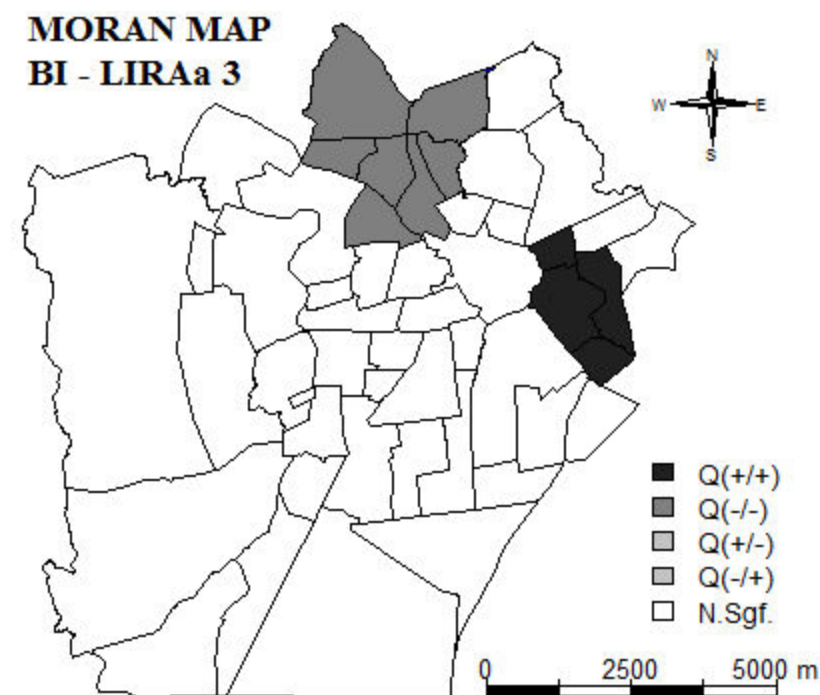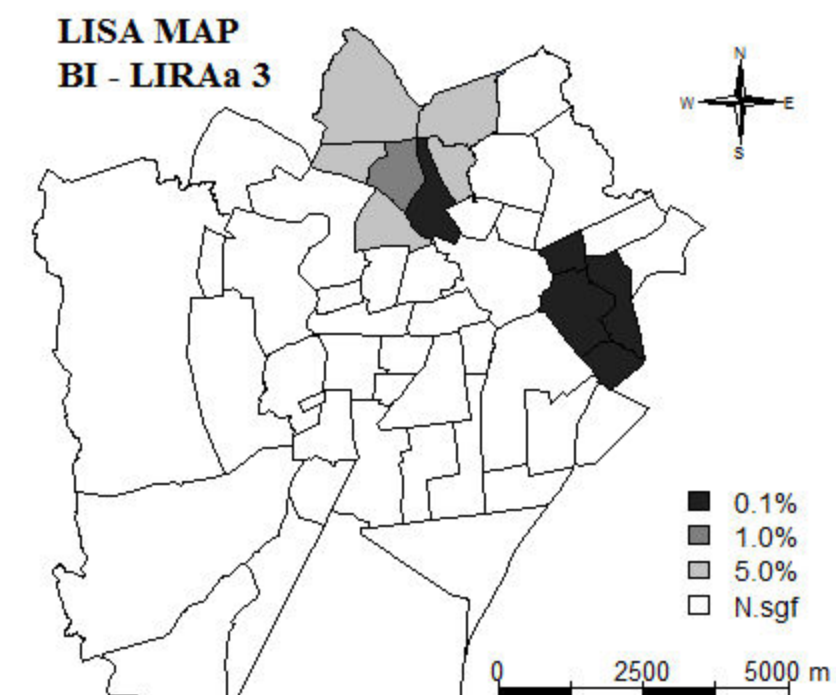

Supplement: Supplementary file 10 — Additional file 10: Figure S10. Moran scatterplots of the BI data, the LISA maps, and the Moran maps in 2016. [file 13071_2020_4070_MOESM10_ESM.pdf]
