# Supplementary material for: Spatial modelling of the infestation indices of Aedes aegypti: an innovative strategy for vector control actions in developing countries
Source: Parasit Vectors. 2020 Apr 16;13:197. doi: 10.1186/s13071-020-04070-w (PMC7164210; doi:10.1186/s13071-020-04070-w)
Supplement: Supplementary file 7 — Additional file 7: Figure S7. Moran scatterplots of the HI data, the LISA maps, and the Moran maps in 2015. [file 13071_2020_4070_MOESM7_ESM.pdf]

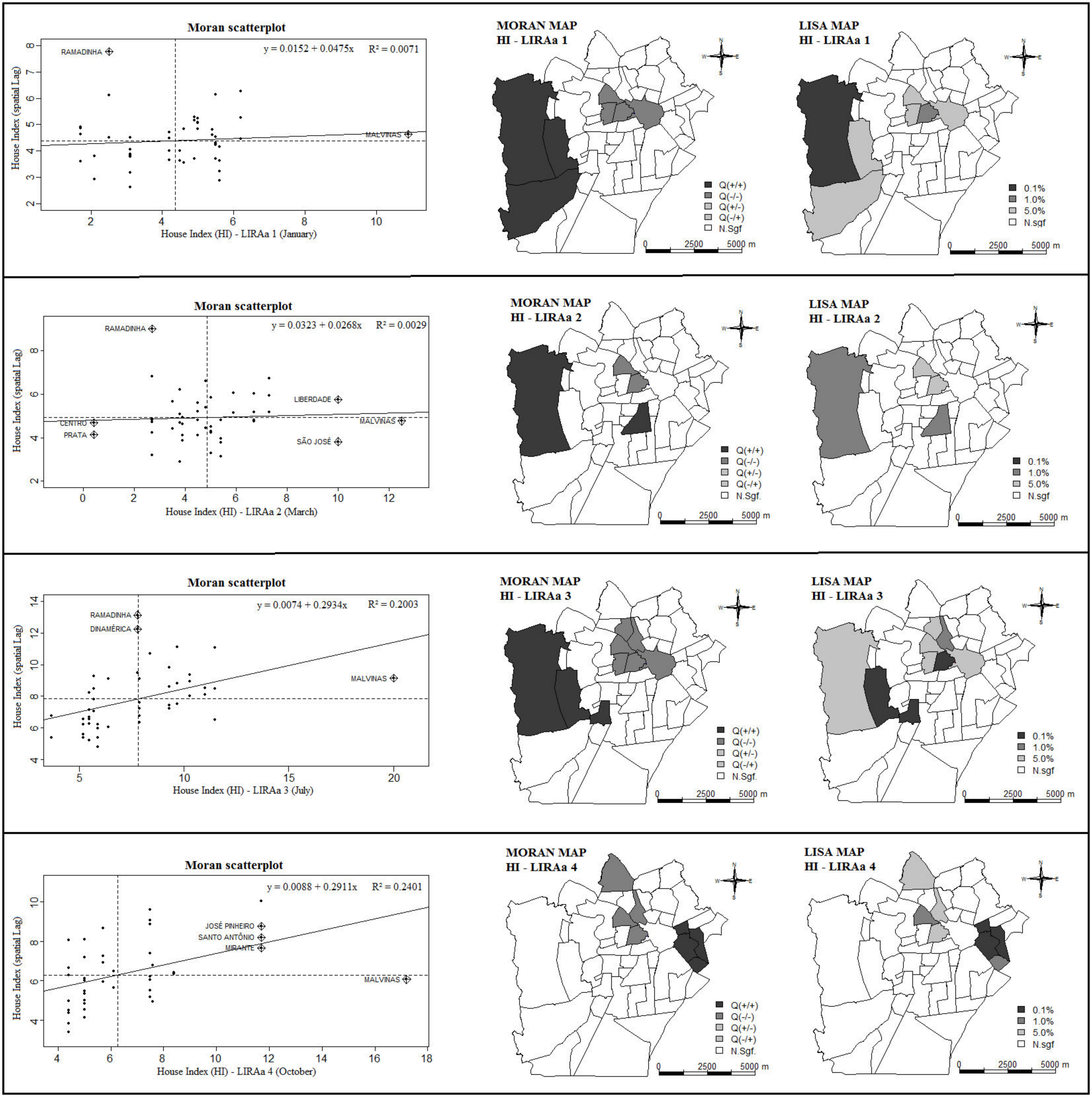

Moran scatterplot
y = 0.0152 + 0.0475x
R² = 0.0071
RAMADINHA
MALVINAS
House Index (spatial Lag)
House Index (HI) - LIRAa 1 (January)
MORAN MAP
HI - LIRAa 1
Q(+/+)
Q(-/-)
Q(+/-)
Q(-/+)
N.Sgf
0 2500 5000 m
LISA MAP
HI - LIRAa 1
0.1%
1.0%
5.0%
N.sgf
0 2500 5000 m
Moran scatterplot
y = 0.0323 + 0.0268x
R² = 0.0029
RAMADINHA
LIBERDADE
CENTRO
PRATA
MALVINAS
SÃO JOSÉ
House Index (spatial Lag)
House Index (HI) - LIRAa 2 (March)
MORAN MAP
HI - LIRAa 2
Q(+/+)
Q(-/-)
Q(+/-)
Q(-/+)
N.Sgf.
0 2500 5000 m
LISA MAP
HI - LIRAa 2
0.1%
1.0%
5.0%
N.sgf
0 2500 5000 m
Moran scatterplot
y = 0.0074 + 0.2934x
R² = 0.2003
RAMADINHA
DINAMÉRICA
MALVINAS
House Index (spatial Lag)
House Index (HI) - LIRAa 3 (July)
MORAN MAP
HI - LIRAa 3
Q(+/+)
Q(-/-)
Q(+/-)
Q(-/+)
N.Sgf.
0 2500 5000 m
LISA MAP
HI - LIRAa 3
0.1%
1.0%
5.0%
N.sgf
0 2500 5000 m
Moran scatterplot
y = 0.0088 + 0.2911x
R² = 0.2401
JOSÉ PINHEIRO
SANTO ANTÔNIO
MIRANTE
MALVINAS
House Index (spatial Lag)
House Index (HI) - LIRAa 4 (October)
MORAN MAP
HI - LIRAa 4
Q(+/+)
Q(-/-)
Q(+/-)
Q(-/+)
N.Sgf.
0 2500 5000 m
LISA MAP
HI - LIRAa 4
0.1%
1.0%
5.0%
N.sgf
0 2500 5000 m
