# Supplementary material for: Spatial modelling of the infestation indices of Aedes aegypti: an innovative strategy for vector control actions in developing countries
Source: Parasit Vectors. 2020 Apr 16;13:197. doi: 10.1186/s13071-020-04070-w (PMC7164210; doi:10.1186/s13071-020-04070-w)
Supplement: Supplementary file 9 — Additional file 9: Figure S9. Moran scatterplots of the HI data, the LISA maps, and the Moran maps in 2016. [file 13071_2020_4070_MOESM9_ESM.pdf]

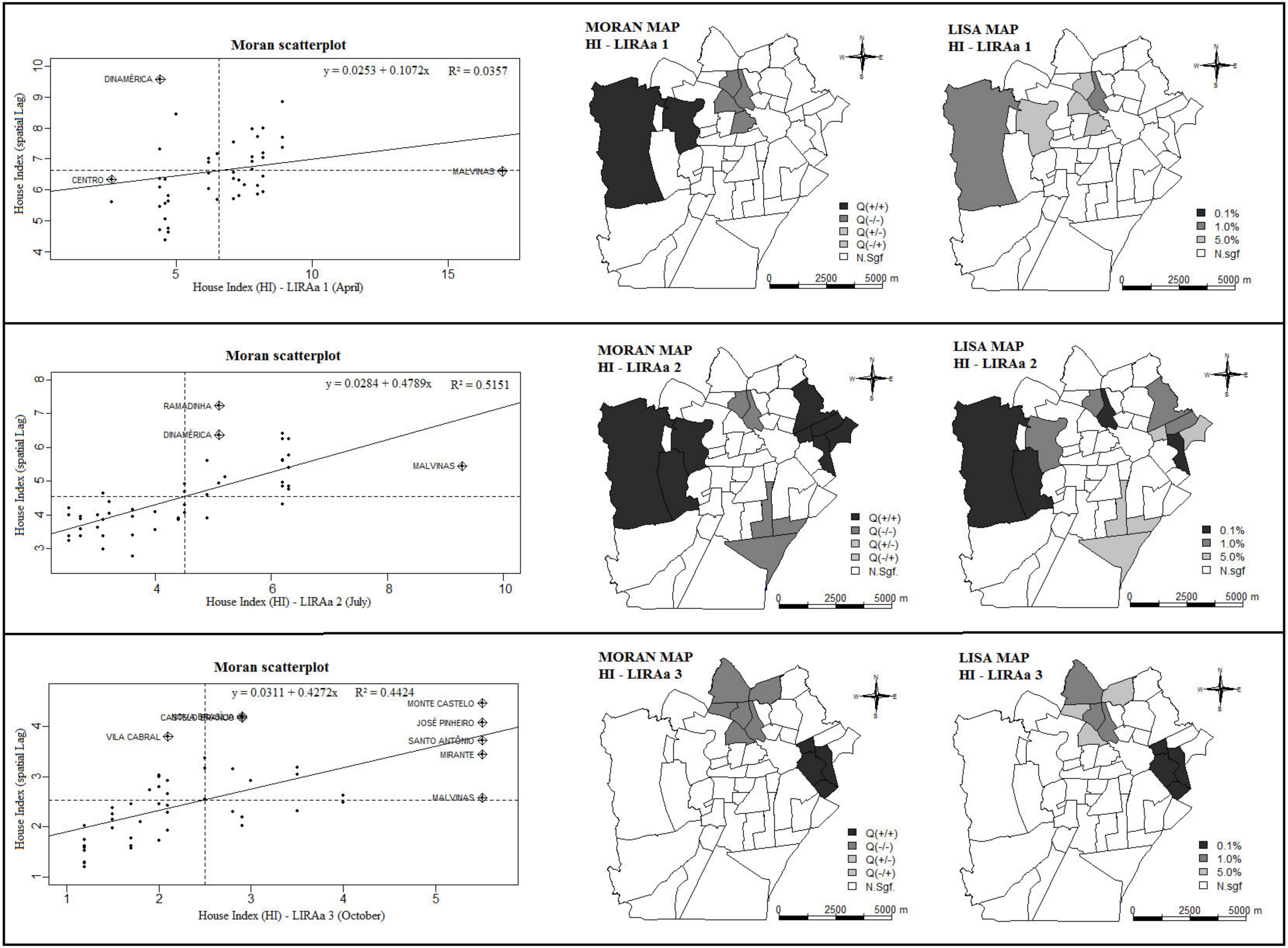

Moran scatterplot
y = 0.0253 + 0.1072x
R² = 0.0357
DINAMÉRICA
CENTRO
MALVINAS
House Index (spatial Lag)
House Index (HI) - LIRAa 1 (April)
MORAN MAP
HI - LIRAa 1
Q(+/+)
Q(-/-)
Q(+/-)
Q(-/+)
N.Sgf
0
2500
5000 m
LISA MAP
HI - LIRAa 1
0.1%
1.0%
5.0%
N.sgf
Moran scatterplot
y = 0.0284 + 0.4789x
R² = 0.5151
RAMADINHA
DINAMÉRICA
MALVINAS
House Index (spatial Lag)
House Index (HI) - LIRAa 2 (July)
MORAN MAP
HI - LIRAa 2
Q(+/+)
Q(-/-)
Q(+/-)
Q(-/+)
N.Sgf.
LISA MAP
HI - LIRAa 2
0.1%
1.0%
5.0%
N.sgf
Moran scatterplot
y = 0.0311 + 0.4272x
R² = 0.4424
MONTE CASTELO
JOSÉ PINHEIRO
SANTO ANTÔNIO
MIRANTE
MALVINAS
VILA CABRAL
House Index (spatial Lag)
House Index (HI) - LIRAa 3 (October)
MORAN MAP
HI - LIRAa 3
Q(+/+)
Q(-/-)
Q(+/-)
Q(-/+)
N.Sgf.
LISA MAP
HI - LIRAa 3
0.1%
1.0%
5.0%
N.sgf
