# Supplementary material for: Spatial modelling of the infestation indices of Aedes aegypti: an innovative strategy for vector control actions in developing countries
Source: Parasit Vectors. 2020 Apr 16;13:197. doi: 10.1186/s13071-020-04070-w (PMC7164210; doi:10.1186/s13071-020-04070-w)
Supplement: Supplementary file 6 — Additional file 6: Figure S6. Moran scatterplots of the BI data, the LISA maps, and the Moran maps in 2014. [file 13071_2020_4070_MOESM6_ESM.pdf]

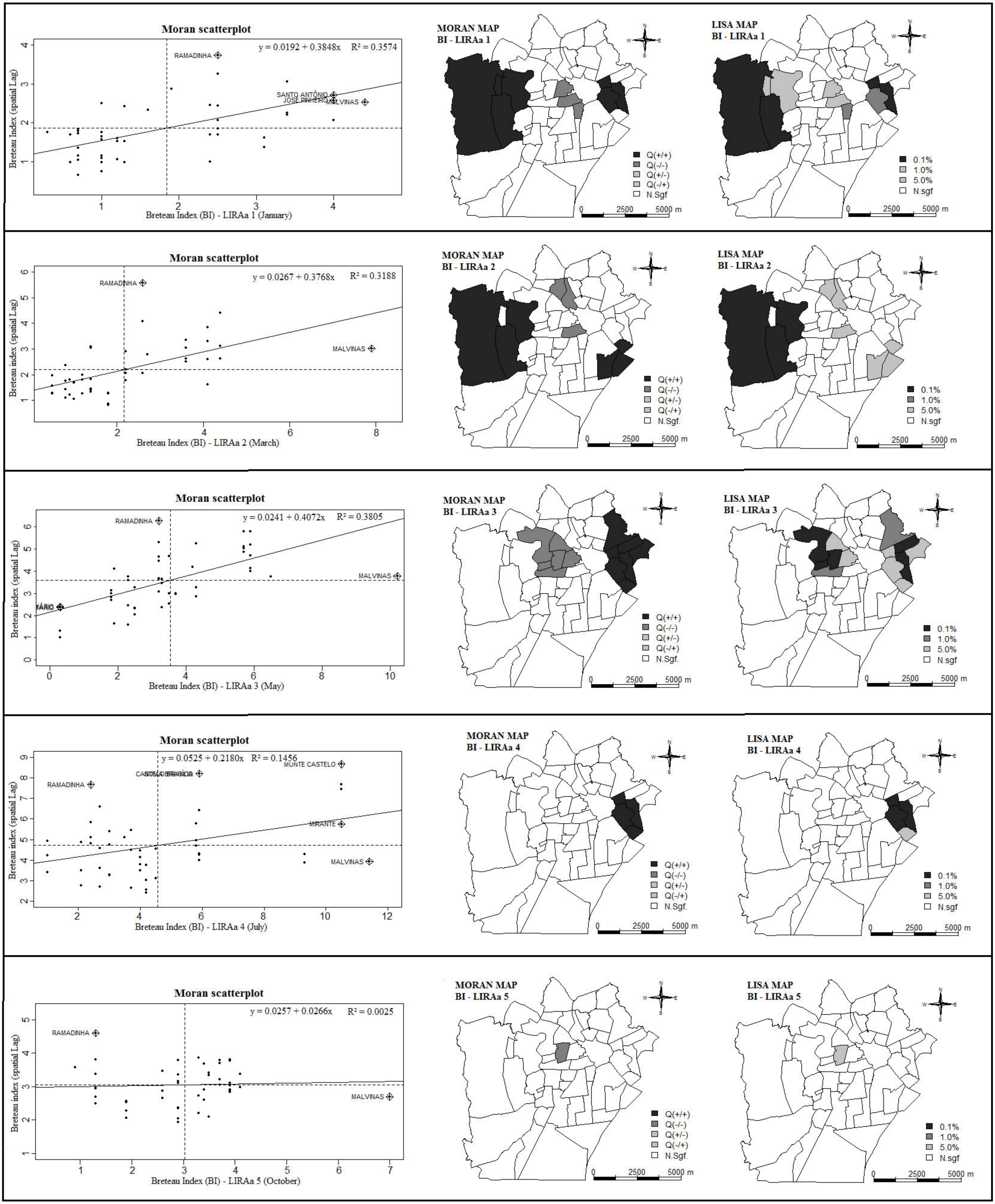

Moran scatterplot
y = 0.0192 + 0.3848x
R² = 0.3574
RAMADINHA
SANTO ANTÔNIO
JOSÉ PINHEIRO
MALVINAS
Breteau Index (spatial Lag)
Breteau Index (BI) - LIRAa 1 (January)
MORAN MAP
BI - LIRAa 1
Q(+/+)
Q(-/-)
Q(+/-)
Q(-/+)
N.Sgf
LISA MAP
BI - LIRAa 1
0.1%
1.0%
5.0%
N.sgf
0 2500 5000 m
Moran scatterplot
y = 0.0267 + 0.3768x
R² = 0.3188
RAMADINHA
MALVINAS
Breteau index (spatial Lag)
Breteau Index (BI) - LIRAa 2 (March)
MORAN MAP
BI - LIRAa 2
Q(+/+)
Q(-/-)
Q(+/-)
Q(-/+)
N.Sgf.
LISA MAP
BI - LIRAa 2
0.1%
1.0%
5.0%
N.sgf
0 2500 5000 m
Moran scatterplot
y = 0.0241 + 0.4072x
R² = 0.3805
RAMADINHA
MALVINAS
Breteau index (spatial Lag)
Breteau Index (BI) - LIRAa 3 (May)
MORAN MAP
BI - LIRAa 3
Q(+/+)
Q(-/-)
Q(+/-)
Q(-/+)
N.Sgf.
LISA MAP
BI - LIRAa 3
0.1%
1.0%
5.0%
N.sgf
0 2500 5000 m
Moran scatterplot
y = 0.0525 + 0.2180x
R² = 0.1456
RAMADINHA
MONTE CASTELO
MIRANTE
MALVINAS
Breteau index (spatial Lag)
Breteau Index (BI) - LIRAa 4 (July)
MORAN MAP
BI - LIRAa 4
Q(+/+)
Q(-/-)
Q(+/-)
Q(-/+)
N.Sgf.
LISA MAP
BI - LIRAa 4
0.1%
1.0%
5.0%
N.sgf
0 2500 5000 m
Moran scatterplot
y = 0.0257 + 0.0266x
R² = 0.0025
RAMADINHA
MALVINAS
Breteau index (spatial Lag)
Breteau Index (BI) - LIRAa 5 (October)
MORAN MAP
BI - LIRAa 5
Q(+/+)
Q(-/-)
Q(+/-)
Q(-/+)
N.Sgf.
LISA MAP
BI - LIRAa 5
0.1%
1.0%
5.0%
N.sgf
0 2500 5000 m
